# New Antifungal Agents with Azole Moieties

**DOI:** 10.3390/ph15111427

**Published:** 2022-11-17

**Authors:** Melissa Martins Teixeira, Diogo Teixeira Carvalho, Emília Sousa, Eugénia Pinto

**Affiliations:** 1 Laboratory of Organic and Pharmaceutical Chemistry, Department of Chemical Sciences, Faculty of Pharmacy, University of Porto, 4050-313 Porto, Portugal; 2 Laboratory of Microbiology, Department of Biological Sciences, Faculty of Pharmacy, University of Porto, 4050-313 Porto, Portugal; 3 Interdisciplinary Centre of Marine and Environmental Research (CIIMAR), University of Porto, 4450-208 Matosinhos, Portugal; 4 Laboratory of Research in Pharmaceutical Chemistry, Department of Food and Drugs, Faculty of Pharmaceutical Sciences, Federal University of Alfenas, Alfenas 37137-001, Brazil

**Keywords:** antifungal drugs, azoles, new developments

## Abstract

Fungal conditions affect a multitude of people worldwide, leading to increased hospitalization and mortality rates, and the need for novel antifungals is emerging with the rise of resistance and immunocompromised patients. Continuous use of azole drugs, which act by inhibiting the fungal CYP51, involved in the synthesis of ergosterol, essential to the fungal cell membrane, has enhanced the resistance and tolerance of some fungal strains to treatment, thereby limiting the arsenal of available drugs. The goal of this review is to gather literature information on new promising azole developments in clinical trials, with in vitro and in vivo results against fungal strains, and complementary assays, such as toxicity, susceptibility assays, docking studies, among others. Several molecules are reviewed as novel azole structures in clinical trials and with recent/imminent approvals, as well as other innovative molecules with promising antifungal activity. Structure–activity relationship (SAR) studies are displayed whenever possible. The azole moiety is brought over as a privileged structure, with multiple different compounds emerging with distinct pharmacophores and SAR. Particularly, 1,2,3-triazole natural product conjugates emerged in the last years, presenting promising antifungal activity and a broad spectrum against various fungi.

## 1. Introduction

Over the last few decades, fungal infections have been considered as a serious concern to the population, with increasing incidence and resistance to the clinically available drugs. Fungi-associated conditions affect more than a billion people worldwide, with more than 150 million being severe, life-threatening conditions, leading to approximately 1.7 million deaths annually (according to a 2017 study) [1,2]. Fungal infections (mycoses) might derive from opportunistic pathogens, such as *Candida* and *Aspergillus* species, the most frequent invasive fungal infection (IFIs) agents, particularly in immunocompromised patients. Dermatophytosis, superficial fungal infections, are also very common and difficult to treat [3]. As the number of immunocompromised patients increases, especially during the COVID-19 pandemic, so does the occurrence of opportunistic fungal-like diseases and infections [4,5].

Although challenging, the search for new antifungal agents increased in the last few years, due to the arise of resistance problems of fungi to the existing therapeutics, new fungal strains and, as previously mentioned, the increased occurrence of fungal infections in the past decades, leading to the approval of new antifungal drugs in 2021 and 2022 [1,6]. Nonetheless, there are still limited options and no breakthroughs in novel scaffolds with three predominant classes in clinical: polyenes, echinocandins, and azoles, currently the most utilized antifungal class [5,7,8]. Drug–drug interactions, associated toxicity, and tolerability issues, as well as previously mentioned problems, drove researchers to search for new therapeutical antifungal agents with a broader spectrum, higher potency, improved safety profile, and fungicidal activity [7,9].

Most used antifungal agents are included in the class of azoles. This family of compounds has shown over the decades a vast range of applications in the treatment of fungal infections and diseases, with increasing interest and use in the clinical setting. Therefore, the development of new promising molecules with an azole scaffold has rapidly emerged throughout the years [7,9]. In this review, new developments focusing on the azole antifungal class of drugs will be discussed, highlighting emerging drug candidates with this chemical scaffold. An historical perspective of antifungal azole drugs and their mechanism of action will be introduced, and new approaches and clinical trial candidates will be presented. When possible, structure–activity relationship (SAR) studies of these newly synthesized compounds will be discussed.

## 2. History and the Evolution of Azoles

**Figure 1:**
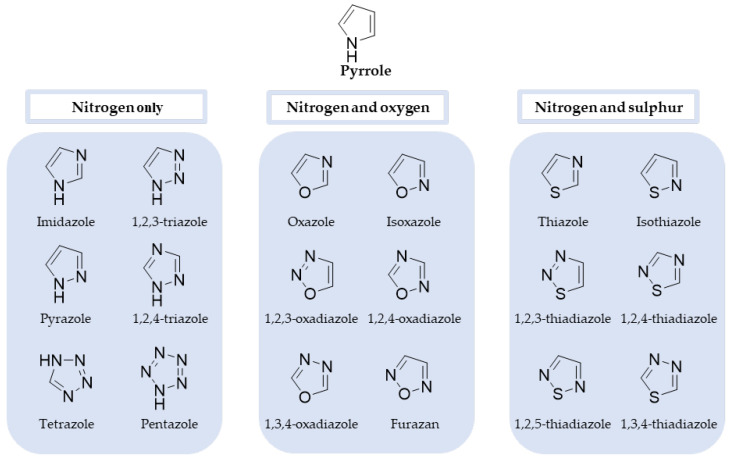
Chemical structure of pyrrole, the simplest azole, as well as azole rings containing nitrogen only, nitrogen and oxygen, and nitrogen and sulphur [10,11].

Azoles are five-membered heterocyclic and aromatic molecules containing at least one nitrogen atom and two double bonds. Additionally, the ring can also have oxygen or sulphur atoms conjugated on the heterocycle, generating different parent structures, as exemplified in Figure 1. Pyrrole, the simplest azole, is also represented [10]. From the azole rings shown, two of them represent major classes for antifungal drugs: imidazole and triazole, which will be discussed later in this review.

Numerous and diverse synthetic approaches to obtain antifungal azoles have been applied and will not be contemplated in this review. For details on the synthesis of azoles, recent reviews can be consulted [12,13,14,15,16].

**Figure 2:**
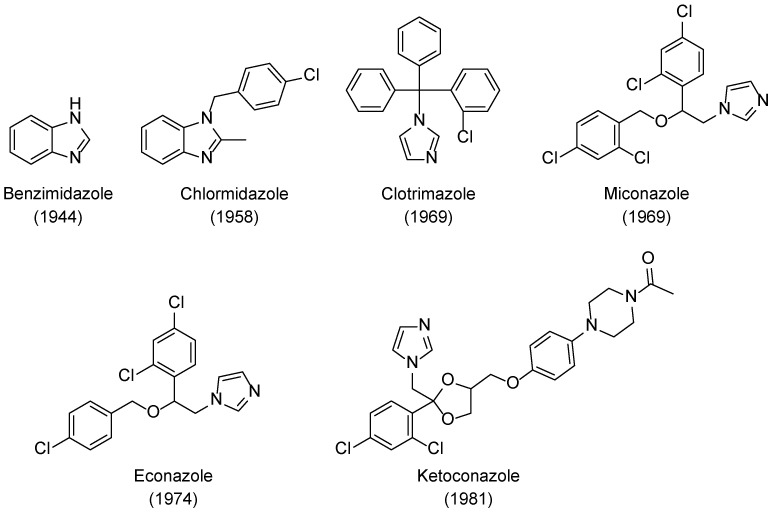
Structures and approval dates of imidazole antifungal drugs [9,17].

Benzimidazole (Figure 2) was the first azole described that presented antifungal activity, by Woolley in 1944 [17]. However, it was not until 1958 that an azole antifungal drug was developed, with the introduction of chlormidazole (Figure 2) to the market, which set off the search for azole compounds for antifungal therapy [9]. Subsequently, new azoles were developed, and three new antifungal azoles surfaced as topical agents: clotrimazole and miconazole appeared in 1969 and econazole short after that, in 1974 (Figure 2). These set of azole drugs, due to similar physiochemical properties and similar structure, containing an imidazole ring, are grouped as first generation imidazoles [9]. Ketoconazole was introduced in 1981 and labelled as a second-generation imidazole (Figure 2). Unlike the previous imidazoles, this compound was not limited to treat superficial mycoses, and it was the first azole orally available to treat systemic fungal infections [18,19,20]. However, ketoconazole presented several side effects, lack of effectiveness, low selectivity, and recurrence of the fungal infections, which motivated the search for new azole derivatives and, later, its removal from the market to treat systemic mycosis [9,19].

**Figure 3:**
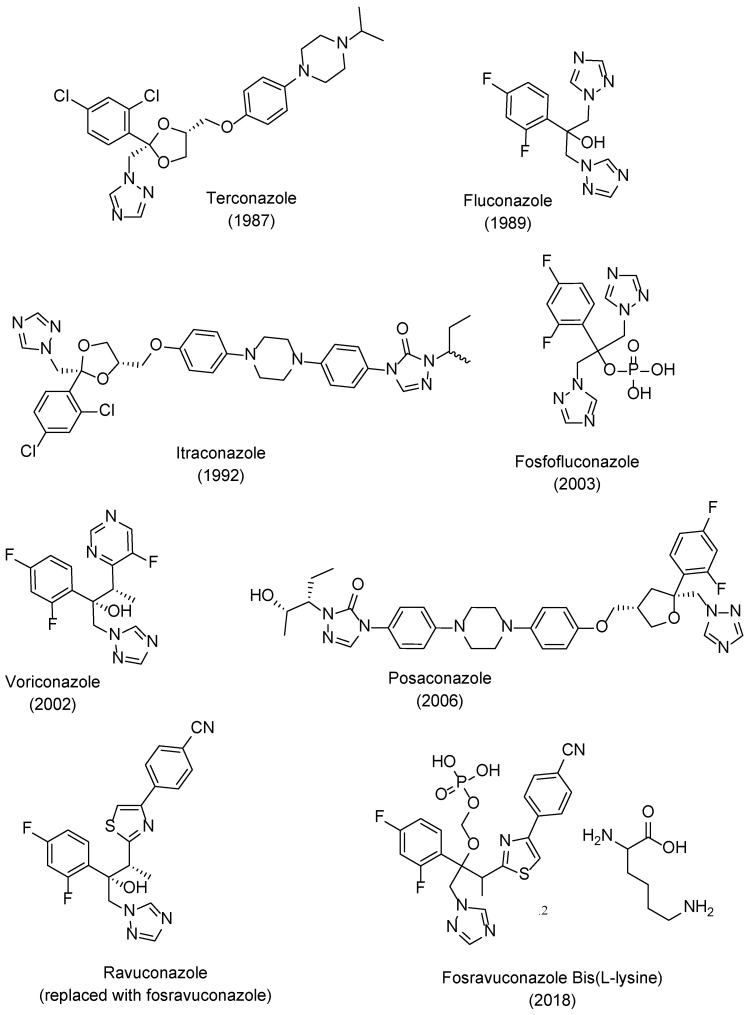
Structures and approval dates of triazole antifungal drugs [9,19,21].

The new class of triazole antifungal drugs (Figure 3) presented various advantages over the previous used imidazoles, such as having suitable solubility, greater affinity to the target fungal enzyme than to the human one and, therefore, higher selectivity and safety, and a broader spectrum of action [18]. Terconazole was the first of this class to be marketed and it belongs to the first generation of triazoles, as well as fluconazole, itraconazole, and fosfluconazole, the latter being a prodrug of fluconazole developed to achieve higher intravenous bolus administration than fluconazole. Fluconazole was approved in 1989 and presented good pharmacokinetics (PK) and a broad spectrum of antifungal action. Due to its safety profile, fluconazole rapidly replaced ketoconazole against various conditions. However, resistance to this drug has emerged in the last few decades. Itraconazole also presented a broad spectrum of action and decreased toxicity profile when compared to ketoconazole [19]. Although this new class of antifungal drugs presented higher safety than the imidazole drug ketoconazole, drug–drug interactions, toxicity, resistance problems, and pharmacokinetic issues were still present, which represented limitations in the clinical aspect [19].

To further improve PK, safety, to broaden the spectrum of antifungal activity, to possibly fight resistant strains and in attempt to obtain fungicidal activity for some species, second generation triazoles emerged. Voriconazole was developed from fluconazole and approved in 2002. Posaconazole, an analog of itraconazole with a wide range of antifungal activity was approved in 2006. Ravuconazole, a derivative of fluconazole, displayed a broad spectrum of antifungal activity and higher potency than fluconazole, which is promising against fluconazole-resistant strains. A prodrug of ravuconazole, fosravuconazole bis(L-lysine), was developed and approved in 2018 in Japan. Other second generation triazoles include albaconazole, efinaconazole, and isavuconazole (structures not shown here) [8,9,19,21]. Currently, there are about 23 antifungal azoles in the market, with clotrimazole, ketoconazole, miconazole, fluconazole, itraconazole, and voriconazole being the most used ones [22].

## 3. Mechanism of Action of Azole Antifungals

Although there are several mechanisms disclosed for antifungal action, among azole drugs all share a common mechanism of action. Antifungal azoles act by targeting the fungal cell membrane, affecting the synthesis of ergosterol. Ergosterol is a mammalian cholesterol derivative, and it is a fundamental component of the fungal cell membrane. Moreover, ergosterol acts as a stimulator of growth and proliferation in fungal cells, due to its hormone-like role. This function could be impaired if ergosterol is depleted and replaced with uncommon sterols [3,9,18].

**Figure 4:**
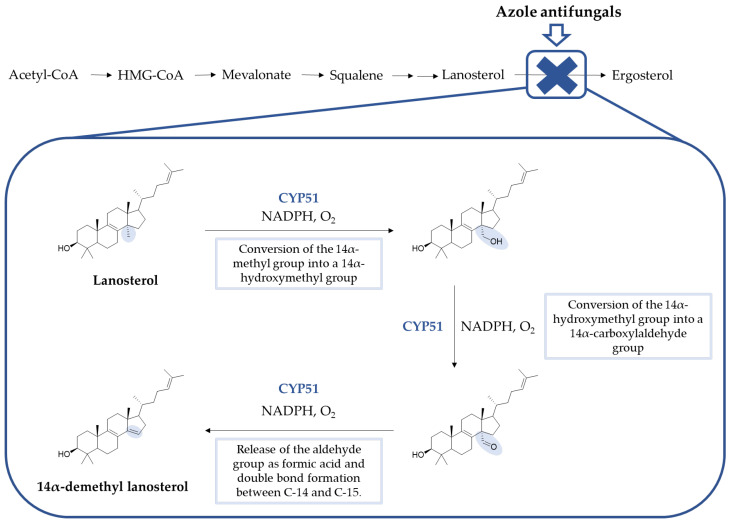
Summarized synthesis of ergosterol, the fungal sterol, and detailed steps of CYP51 conversion of lanosterol to 14α-demethyl lanosterol (Adapted from Peyton, 2015) [8,23].

Azoles inhibit the lanosterol 14-α-demethylase (or CYP51), a fungal cytochrome P450-dependent enzyme responsible for the transformation of lanosterol in 14-demethyl lanosterol in the ergosterol biosynthetic pathway. CYP51 catalyzes the conversion of the 14-α-methyl group on lanosterol to 14-α-hydroxymethyl and, posteriorly, to 14-α-carboxyaldehyde. This group is released as formic acid, and leads to the introduction of a double bond between C-14 and C-15 (Figure 4) [8]. By inhibition of this enzyme, the levels of lanosterol and 14-α-methylsterols increase and the levels of ergosterol decrease. This results in an alteration of the normal permeability and fluidity of the fungal cell membrane, which will lead to consequences to the activity of membrane-bound enzymes and inhibition of growth and replication of fungal cells [18,19,20]. In some fungal species, azoles can also inhibit the subsequent ∆^22^-desaturase step [23]. Additionally, against *Candida* species, azoles, such as voriconazole and miconazole, presented fungicidal activity, the latter associated with the accumulation of reactive oxygen species (ROS) in biofilm cells [24].

**Figure 5:**
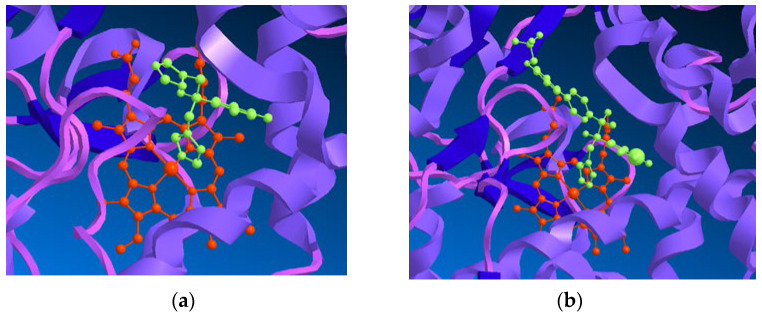
(**a**) Representation of the interaction of fluconazole (in green), a known triazole antifungal drug, with the heme group (in red) in the active site of CYP51 from *Saccharomyces cerevisiae*; (**b**) Representation of the interaction of VT-1161 (in green), a tetrazole antifungal agent, with the heme group (in red) in the active site of CYP51 from *Candida albicans*. ChemBio3D was used to visualize (PDB 4WMZ and PDB 5TZ, respectively) [25,26].

The CYP51 enzyme presents a protoporphyrin moiety, with an iron atom situated at the active site, to which the azole antifungals bind through one of the nitrogen atoms located on the azole ring. Depending on the remaining structure, other interactions with the target molecule are established, which will determine the target and azole conformation, as well as the affinity and selectivity for the fungal enzyme. As previously mentioned, the earlier series of azole antifungals bearing an imidazole ring were replaced with a triazole ring, which presented higher specificity to the fungal cytochrome P450 and better safety profile [19,23]. This enzyme also catalyzes the synthesis of cholesterol in mammals. For an antifungal to be efficient and safe, it must demonstrate higher affinity (and, therefore, higher specificity) to the fungal CYP51 instead of the mammalian one [18]. Inhibition of other enzymes of the cytochrome P450 involved in the biosynthesis of cholesterol can result in toxicity and drug–drug interactions, resulting in less than ideal selectivity for the fungal enzyme [19]. A visual representation for the interaction of fluconazole, a known triazole antifungal, and VT-1161, a novel tetrazole antifungal agent, with the active site of CYP51 is shown in Figure 5.

## 4. New Antifungal Azoles in Research and Development

Continuous use of azoles may cause resistance problems (acquired resistance), which can be correlated with mutations on the target enzyme, namely of the genes that encode CYP51, increased expression of these genes, or overexpressed efflux by membrane pumps (that will expel the drug from the cell) [23,27,28]. Moreover, some fungal strains might be intrinsically resistant, both by weak affinity of the drug to the target molecule and/or enhanced efflux [27]. Additionally, widespread use of antifungal agents in the prophylaxis or treatment of IFIs has led to the development of clinical resistance, which represents a burden to researchers due to its undetectable nature in in vitro susceptibility testing and in vivo animal models [29]. Therefore, due to their relevance in the clinic, a continuous investigation to improve azoles efficacy to overcome drug resistance with optimized derivatives and even new approaches of hybridization is being followed.

### 4.1. Azoles in Clinical Trials

#### 4.1.1. Luliconazole

**Figure 6:**
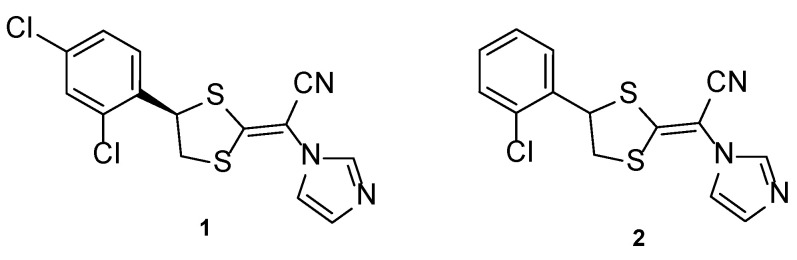
Chemical structures of luliconazole (**1**) and lanoconazole (**2**) [30].

Luliconazole, or NND-502 (**1**, Figure 6), is a novel vinyl-imidazole antifungal agent of topical use and a follow-up candidate of lanoconazole (**2**, Figure 6), an imidazole compound with a ketene dithioacetal moiety which has shown antifungal activity against a variety of fungal strains as the *R*-enantiomer (with stronger potency than the racemate). Similarly, the structurally related *R*-enantiomer of luliconazole (**1**) displayed higher antifungal potency than the racemic compound, and stronger activity than lanoconazole (**2**) [30].

Luliconazole (**1**) was tested using the broth microdilution method to measure the antifungal activity against *Trichophyton* species, using lanoconazole (**2**) as standard and expressing the obtained results in MIC, the minimal concentration required to inhibit fungal growth. In this study, luliconazole (**1**) presented lower concentrations of MIC (ranging from 0.0018 to 0.056 µM) than lanoconazole (**2**) (MIC values from 0.0041 to 0.125 µM), therefore demonstrating its antifungal potential. To evaluate the in vivo activity of this compound, Niwano et al. utilized the guinea pig model, and the compound and standard drugs were applied topically. When compared to the standard drugs used, luliconazole (**1**) presented promising results and strong antifungal activity, since all infected guinea pigs feet became fungus-free (although the standards presented high antifungal potency, results for luliconazole (**1**) were stronger and therefore making it an optimal drug candidate). It is important to note the fungicidal activity of this compound **1**, which makes it an interesting antifungal agent [31].

Antifungal activity against *Candida albicans* and *Aspergillus fumigatus* was also tested in vitro using the broth microdilution method and in vivo using murine models and compared to the standards fluconazole and itraconazole. Against *C. albicans*, luliconazole (**1**) displayed lower concentrations of MIC (ranging from 0.087 to 0.706 µM) than fluconazole (MIC values ranging from 0.42 to 1.63 µM), which demonstrated higher antifungal potency. Against *A. fumigatus*, luliconazole (**1**) demonstrated MIC values of ≤0.00087 µM, lower than those of itraconazole (MIC ~ 0.00089 µM). In vivo studies revealed that oral luliconazole (**1**) was less promising in terms of increasing survival rates of mice than fluconazole against *C. albicans*, and that against *A. fumigatus* opposite results were observed, as luliconazole (**1**) revealed to prolongate the survival when compared to the standards fluconazole and itraconazole. Due to the observed results, additional in vivo studies using a rat model were performed and were in accordance with in vitro results for the *Aspergillus* strain, making this compound an interesting oral antifungal agent for the treatment of aspergillosis [32].

Luliconazole (**1**) formulated as a 1% cream was firstly approved in Japan in 2005, following its approval in India in 2009 and in the United States in 2013 for the treatment of tinea infections. Clinical trials for luliconazole (**1**) are presented in Table 1 [30]. In the future, clinical trials to treat *Candida* infections would be beneficial to support in vitro studies and broaden the clinical spectrum of this promising antifungal agent.

#### 4.1.2. Isavuconazole

**Figure 7:**
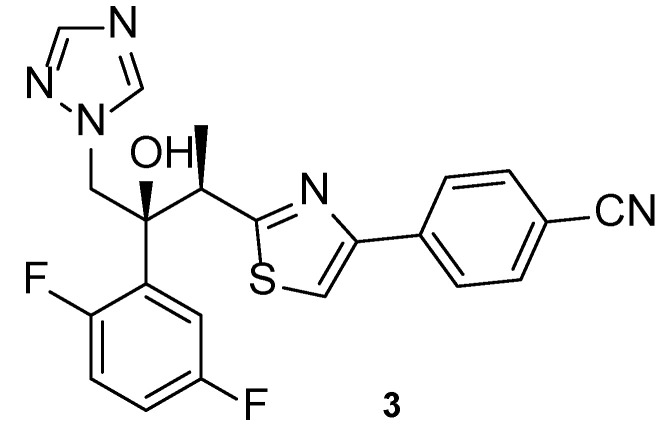
Chemical structure of isavuconazole (**3**), a novel triazole antifungal agent [41].

Isavuconazole, or BAL4815 (**3**, Figure 7), a 2nd generation triazole antifungal drug, was approved in 2015 in the United States to treat mucormycosis and invasive aspergillosis. Ever since then, this azole agent has been used in the prophylaxis of IFIs and to treat infections caused by other fungal strains due to its broad spectrum of action, which has been demonstrated by several studies. This compound is administered orally or by intravenous route as isavuconazonium sulfate, a prodrug that releases isavuconazole (**3**) in vivo and has a high volume of distribution and bioavailability [40].

Isavuconazoles’ antifungal activity against *Candida*, *Aspergillus*, Mucorales, *Fusarium,* and *Scedosporium* has been studied. When tested against *Aspergillus* species, isavuconazole (**3**) presented promising antifungal results, with MIC values similar/comparable to those of voriconazole [41]. Additionally, when tested against azole-resistant *Aspergillus lentulus*, isavuconazole (**3**) displayed promising activity [42]. Against Mucorales species, which was little susceptible to the standard voriconazole, isavuconazole (**3**) displayed little antifungal activity. Moreover, isavuconazole (**3**) revealed moderate activities against *Fusarium* and *Scedosporium* species and displayed comparable activity to the standards fluconazole, voriconazole and itraconazole against *Candida* species, being efficient even to fluconazole-resistant *Candida krusei* [41,43]. Further investigation on the in vitro antifungal activity of isavuconazole (**3**) should be performed for *Fusarium*, *Scedosporium*, and Mucorales species, to confirm the obtained results.

In terms of in vivo results, a phase 3 clinical trial (NCT00412893, Table 2), with published results in 2016, studied the efficacy and safety of isavuconazole (**3**) in comparison to voriconazole, in patients with suspected invasive aspergillosis [43]. Both azoles demonstrated similar, successful results, with isavuconazole (**3**) causing fewer side effects than voriconazole [43]. Similar effects were found by Bongomin et al. in 2019, when the same drugs were assessed for the treatment of chronic pulmonary aspergillosis and adverse effects were compared [44]. As previously mentioned, the use of isavuconazole (**3**) was approved for the treatment of mucormycosis, which is a fungal disease with high mortality rates and little treatment options. Prior to the approval, a phase 3 study (NCT00634049, Table 2) was performed with the purpose of assessing the efficacy and safety against invasive aspergillosis and rare disease-causing fungi such as Mucorales. When comparing the effects of isavuconazole (**3**) and amphotericin B against patients with mucormycosis, similar responses were obtained, with isavuconazole (**3**) presenting high tolerability and effectiveness [45]. Other studies confirmed these findings [40]. Intravenous isavuconazole (**3**) was tested in a phase 3 clinical trial (NCT00413218, Table 2) vs. intravenous caspofungin followed by the testing of oral isavuconazole (**3**) vs. oral voriconazole for the treatment of invasive candidiasis, which is a growing disease, causing hospitalizations and mortality in patients. Patients with candidemia or invasive candidiasis received the intravenous drugs for 10 days, with successful outcomes for both the tested drugs. When followed by the oral drugs, the use of isavuconazole (**3**) presented higher success than voriconazole, which represents promising results for the use of this triazole for the treatment of candidiasis [46]. Isavuconazole (**3**) has an extensive list of clinical trials, some of which are still active, ongoing, and which will not be discussed. Additional information about animal and clinical data can be found in recent literature [47].

#### 4.1.3. SUBA-Itraconazole

SUBA (“super-bioavailability”)-itraconazole is an orally administered azole, which appeared to improve the bioavailability and interpatient variability of the existing antifungal drug, itraconazole [51].

In a phase 3 study to assess the safety and tolerability of SUBA-itraconazole when used in IFI prophylaxis (NCT03572049, Table 3), patients receiving stem cell transplants were treated with the tested compound vs. the conventional itraconazole. Resulting data showed that the novel formulation led to faster achievement of therapeutic levels, with less variability, no gastrointestinal toxicity and, therefore, higher tolerability and safety profile and increased bioavailability. Moreover, it is thought to be more cost-effective, due to decreased dosing regimens. Further testing is necessary to prove these results with a larger population and compared to other used drugs in the treatment of IFI prophylaxis [52]. SUBA-itraconazole was approved in the United States by the Food and Drug Administration (FDA) for the treatment of blastomycosis, histoplasmosis, and aspergillosis (when treatment with amphotericin B is not possible) [53].

#### 4.1.4. Iodiconazole

**Figure 8:**
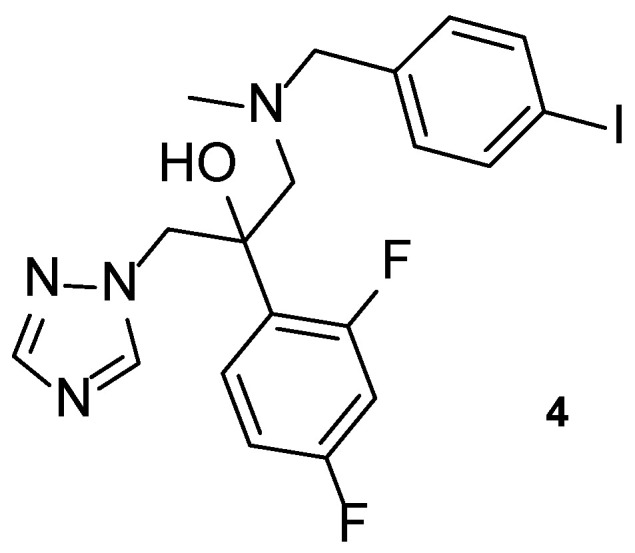
Chemical structure of iodiconazole (**4**), a novel triazole antifungal [55].

Iodiconazole (**4**, Figure 8) is a novel triazole antifungal agent in research, which has demonstrated a broad spectrum of antifungal activity and particular interest against *Aspergillus* species [55]. In 2013, Sun et al. tested iodiconazole (**4**) for in vitro activity against different fungi using the broth microdilution method, and additional in vivo studies were performed to assess PK and correlate with pharmacodynamics data. In order to assess the PK properties of this compound **4**, microdialysis was performed in rats following the determination of the relative recovery (R_in vivo_) and before the administration of iodiconazole (**4**). In terms of pharmacodynamics data, MIC values were calculated in vitro using several azole agents as standards (fluconazole, itraconazole, ketoconazole, and miconazole). By analyzing the obtained results, iodiconazole (**4**) demonstrated promising results against *C. albicans*, *Candida parapsilosis*, *Nannizzia gypsea*, *Microsporum canis*, *Trichophyton violaceum*, *Trichophyton mentagrophytes*, and *Epidermophyton floccosum*, with MIC values ranging from <0.129 to 0.258 µM, lower than those presented for the standard drugs, meaning stronger antifungal potency against these fungal strains [56].

Recently, in 2021, iodiconazole (**4**) was tested in vivo as a topical agent to treat superficial fungal infections, to assess PK properties, dose-response after one dosage and the bioavailability of different formulations (1%, 2%, and 4%) using the tape-stripping method in healthy human volunteers. The results from this study were consistent with previous in vivo studies performed by Wu et al. and will be useful for future research and use of the tested formulations of topical iodiconazole (**4**) [55].

**Figure 9:**
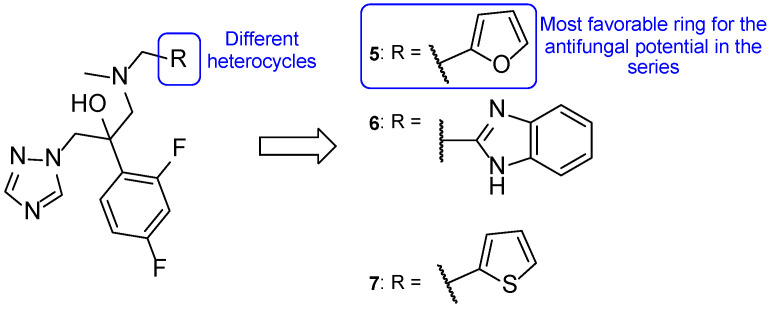
Chemical structures of compounds **5**, **6**, and **7**, novel triazole agents derived from iodiconazole (**4**), and respective SAR [57].

Iodiconazole (**4**) was used as the lead for the design of novel bioisosteric triazole molecules in order to improve its low oral bioavailability and water solubility. The phenyl ring on the side chain was replaced with other heterocycles synthesizing a new series of triazole molecules and attempting to create an oral agent with improved PK properties. The antifungal activity of the compounds was assessed using the broth microdilution method against *C. albicans*, *Candida tropicalis*, *Cryptococcus neoformans*, *Trichophyton rubrum,* and *A. fumigatus*, using fluconazole as standard and displayed results as MIC_80_, defined by the authors as the concentration needed to inhibit 80% of fungal growth. Compound **5** (Figure 9) was the most promising of the compounds derived from iodiconazole (**4**) against *C. albicans*, with a MIC_80_ value of 0.179 µM, lower than the standard (MIC_80_ = 0.816 µM). When tested against *C. tropicalis*, all tested compounds were promising and with MIC values lower than fluconazole (MIC_80_ = 13.06 µM). From the series, compound **5** presented the strongest antifungal activity with a MIC_80_ = 0.046 µM. Except for compound **6** (Figure 9), the tested molecules presented stronger antifungal activity than the standard fluconazole (MIC_80_ values ranging from 0.044 to 0.696 µM vs. 6.53 µM for fluconazole) against *C. neoformans*, with compound **7** (Figure 9) being the strongest one (MIC_80_ = 0.044 µM). Against *A. fumigatus*, to which fluconazole is inactive, compound **7** demonstrated promising antifungal potential (MIC_80_ = 44 µM) and compound **5** showed moderate activity (MIC_80_ = 184 µM). Lastly, against *T. rubrum*, none of the compounds demonstrated promising antifungal activity [57].

From the obtained results, the authors concluded that compound **5** demonstrated the most promising results and that the furan heterocycle in this compound was favorable for the antifungal potential. Moreover, other substituents, such as thiophene (**7**) and pyridine rings, were also favorable for the activity and preferred to the benzimidazole (**6**) and quinoline groups. In addition, LogP values of the series of compounds showed to be adequate for new antifungal oral agents and docking studies were performed for compound **5**, which indicated that the heterocyclic substitution maintained the binding activity of the molecules to the targets’ active site [57].

#### 4.1.5. Albaconazole

**Figure 10:**
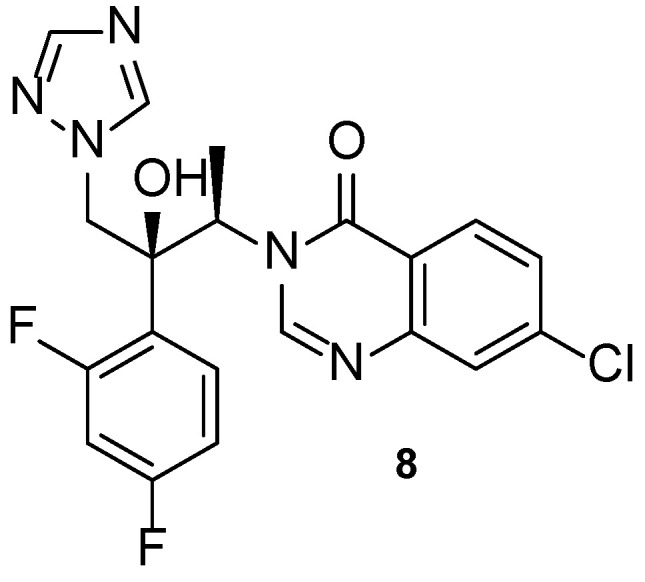
Chemical structure of albaconazole (**8**) [5].

Albaconazole (**8**, Figure 10) is a 2nd generation triazole with a quinazolinone scaffold in its structure, orally administrated and with a broad spectrum of action [5]. A phase 2 study was completed to evaluate the efficacy and safety of oral albaconazole (**8**) for the treatment of onychomycosis of the toenail (NCT00730405, Table 4). Results showed that albaconazole (**8**) was efficient, with higher success than placebo, and proved this compound to have high cure rates and good tolerability, being an alternative for terbinafine and itraconazole treatments [58].

**Figure 11:**
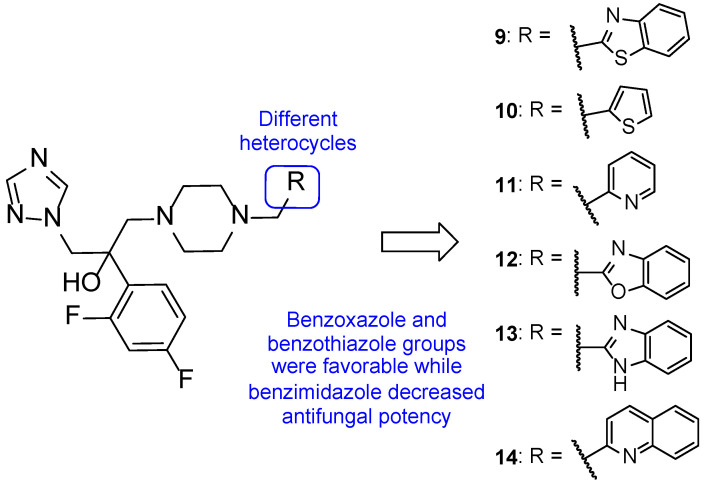
Chemical structures for compounds **9**–**14**, novel triazoles derived from albaconazole (**8**), and respective SAR [57].

In 2013, Jiang et al. designed novel triazole molecules (**9**–**14**, Figure 11) derived from the antifungal drug albaconazole (**8**) by bioisosterically replacing the phenyl ring on the side chain for heterocyclic rings. The goal of this substitution was to create orally available antifungal agents with appropriate PK. The antifungal activity of this new series of compounds was studied using the broth microdilution method against *C. albicans*, *C. tropicalis*, *C. neoformans*, *T. rubrum*, and *A. fumigatus*, using fluconazole as standard. When tested against *C. albicans*, compound **9** presented the lowest MIC_80_ value (0.034 µM), while compounds **10**, **11**, and **12** showed MIC_80_ values of 0.149, 0.151, and 0.138 µM, respectively (higher antifungal potential than fluconazole, with MIC_80_ = 0.816 µM). All tested compounds demonstrated promising antifungal activity against *C. tropicalis*, with MIC_80_ values ranging from 0.009 to 2.41 µM vs. MIC_80_ = 13.06 µM for the standard. The whole series showed promising antifungal potential against *C. neoformans* and *T. rubrum* (MIC_80_ values ranging from 0.016 to 2.4 µM), except for **13** against *C. neoformans* (MIC_80_ = 35.3 µM) and **10** and **13** against *T. rubrum* (MIC_80_ = 9.5 and 141.1 µM, respectively). Compound **9** showed the highest antifungal potential of the series against these strains, with MIC_80_ values of 0.034 and 0.133 µM against *C. neoformans* and *T. rubrum*, respectively. Against *A. fumigatus*, to which fluconazole is intrinsically resistant, compound **12** and **14** showed excellent antifungal activity (MIC_80_ = 0.55 and 4.3 µM, respectively). The remaining compounds demonstrated promising activity, except for **13**. From the results obtained, it was concluded that compounds **9** and **12** were the most promising ones, with a broad spectrum of activity on the tested fungal strains. The isomers of compound **9** were studied and it was noted that the (−)-isomer presented higher antifungal activity than the (+)-isomer. Additionally, SAR studies revealed that benzoxazole (**12**) and benzothiazole (**9**) were favorable for the antifungal activity, while benzimidazole (**13**) resulted in a decrease of antifungal potency. Docking studies of compound **9** demonstrated that the substitution did not affect the potency of the binding to the CYP51 target, although it was a different interaction than albaconazole (**8**), the lead structure. This compound was also evaluated for PK properties using an in vivo study with rats using iodiconazole (**4**) as standard, which revealed better oral absorption, meaning that the goal of the design was successful. Further evaluations are necessary to confirm these results [57].

**Figure 12:**
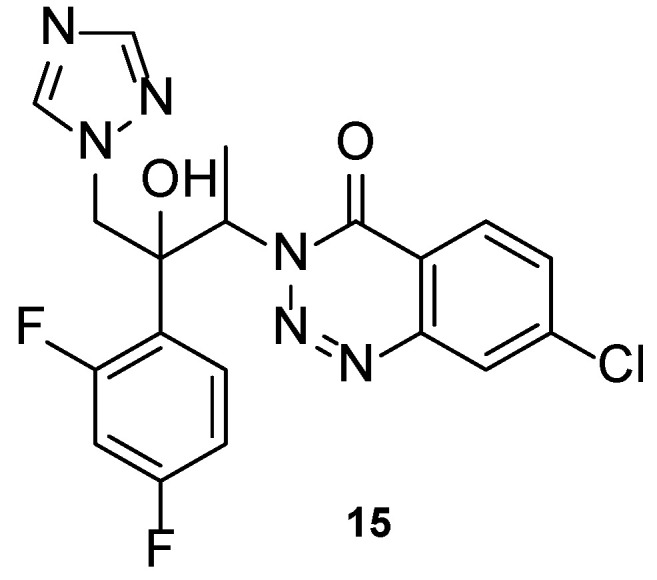
Chemical structure for compound **15**, novel triazole molecule designed based on albaconazole (**8**) [60].

In 2020, novel triazole compounds were designed based on albaconazole (**8**) and tested using the broth microdilution method against *C. albicans*, *C. neoformans*, *A. fumigatus*, and *N. gypsea*. All tested compounds showed good activity against the tested strains, with particular interest regarding the activity of some compounds against *A. fumigatus*. Compound **15** (Figure 12) presented promising results against all fungal species assessed, with MIC values ranging from 0.036 to 0.58 µM (comparable to albaconazole (**8**), with MIC ranging from 0.036 to 2.3 µM) [60].

Selected compounds from the series (structures not shown) were tested against two fluconazole-resistant strains of *C. albicans*. Compound **15** displayed promising antifungal activity (MIC = 18.5 and 4.6 µM), with comparable results to albaconazole (**8**) (MIC = 18.5 and 9.3 µM). SAR studies revealed that substitutions at the C-7 were favorable for the antifungal potency, when compared to substitutions at C-5, C-6, and C-8. From the substitutions at C-7, halogen groups presented higher antifungal activities than electron-withdrawing substituents such as nitro and trifluoromethyl. Compound **15**, with a chlorine substituent at C-7, was the most promising of the series tested. Docking studies performed with compound **15** to the active site of CYP51 in *C. albicans* and *A. fumigatus* revealed similar binding interactions for both strains, but further investigation is needed for lead optimization [60].

#### 4.1.6. PC945

**Figure 13:**
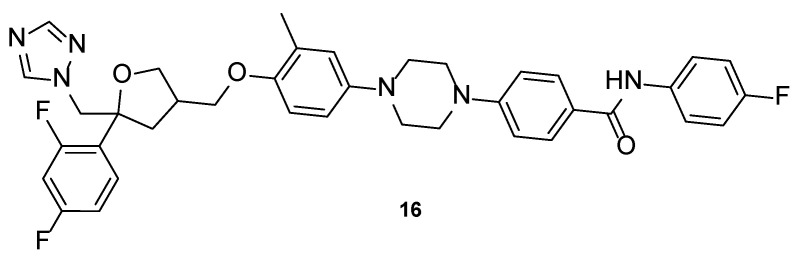
Chemical structure of PC945 (**16**), a novel triazole molecule [62].

Compound PC945 (**16**, Figure 13) is a novel antifungal agent, with a triazole scaffold and characteristics that allow nasal administration. It has been demonstrated that this new triazole molecule has potent antifungal potential. PC945 (**16**) presented a broad spectrum of activity against azole-susceptible and resistant strains of *A. fumigatus*, with MIC values from 0.047 to 11.72 µM, lower/comparable to voriconazole (MIC from 0.183 to 11.45 µM) and comparable to posaconazole (MIC from 0.023 to 2.85 µM). Against *C. albicans*, *Candida glabrata*, and *C. krusei*, this compound showed strong inhibition of fungal growth, with MIC values ranging from 0.119 to 12.08 µM (stronger than voriconazole, with MIC values from 0.4 to 28.6 µM, and comparable to posaconazole, with MIC values from 0.116 to 11.6 µM). When tested against *C. neoformans*, PC945 (**16**) presented equal antifungal potential that voriconazole and posaconazole (MIC = 0.023 µM vs. MIC = 0.023 µM to the standards), and against *Cryptococcus gattii* the antifungal activity was similar to voriconazole and posaconazole (MIC = 0.37 µM vs. MIC = 0.359 and 0.713 µM to voriconazole and posaconazole, respectively). Other strains were tested but with minor results [61].

Additionally, an in vitro model of human alveolus was used to study the synergic effect of PC945 (**16**) and posaconazole or voriconazole against azole-susceptible and resistant *A. fumigatus* strains. Results showed that combination of the known triazoles with the novel antifungal agent was beneficial to the antifungal activity when compared to the use of the drugs alone [63]. PC945 (**16**) has been tested as an intranasal administration in vivo using biomarkers in mice, infected with *A. fumigatus* and using posaconazole and voriconazole as standards. Fungal susceptibility tests were performed and revealed the high susceptibility of the used *A. fumigatus* strain to PC945 (**16**), equal to that of posaconazole, and higher than voriconazole. Results from the intranasal administration of the drugs in mice revealed the high antifungal potential of PC945 (**16**), which inhibited infection successfully and to a larger scale than the standards [64].

Nonclinical and clinical PK properties and the results of a phase 1 clinical trial (NCT02715570, Table 5) for patients with pulmonary aspergillosis, assessing safety and tolerability of this novel triazole agent, were posted recently in 2021. Study results revealed appropriate PK properties, with good drug delivery to the lungs, minimizing side effects, drug–drug interactions, enhancing the local efficacy and rapid absorption to the systemic circulation. In vivo results in humans were concordant with the nonclinical results [65]. The clinical trials available for PC945 (**16**) are summarized in Table 5.

#### 4.1.7. Tetrazole-Pyridine Hybrids

**Figure 14:**
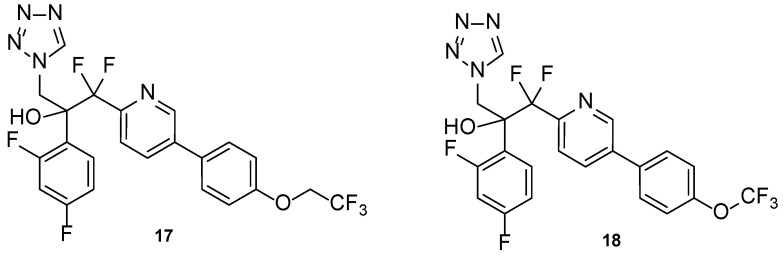
Chemical structures of VT-1161 (**17**) and VT-1129 (**18**) [72].

Hoekstra et al., in 2014, designed and synthesized a series of tetrazole-pyridine hybrids, searching for new, more selective antifungal azoles. The compounds were then tested for in vitro antifungal activity against *C. albicans* and *T. rubrum*, and for selectivity for the fungal CYP51. All compounds presented good antifungal activity, with MIC values from ≤0.0019 to 0.0076 µM (compared to the standard drug itraconazole, with MIC values of 0.023 and 0.088 µM against *C. albicans* and *T. rubrum*, respectively). Compounds **17** and **18** (Figure 14), which presented high in vitro safety, and high selectivity against the fungal enzyme CYP51, proceeded to several more studies [72].

Compound VT-1161, or oteseconazole (**17**, Figure 14), is a tetrazole-pyridine hybrid that has shown promising results in antifungal therapy. In 2014, Warrilow et al. tested its antifungal potential against *C. albicans* (specifically, its potency and selectivity to the fungal CYP51, when compared to the human enzyme), using an array of techniques, including the broth microdilution method, and four standard drugs (clotrimazole, fluconazole, itraconazole, and voriconazole). Oteseconazole (**17**) showed a strong bound to the fungal CYP51, with a dissociation constant (K_d_) ≤39 nM, comparable to the standards used (K_d_ ranging from 10 to 56 nM). Considering the human CYP51, oteseconazole (**17**) displayed no visible binding at the concentration of 86 µM. This is an important observation since binding studies to the human CYP51 are essential to study the drug selectivity and so low toxicity was found. This selectivity study was performed since high antifungal potential was previously demonstrated. When tested for antifungal activity, compound **17** presented great results, with MIC values of 0.0038 µM (compared with MIC ranging from 0.011 to 3.27 µM for fluconazole and voriconazole). According to the obtained results, oteseconazole (**17**) displayed high selectivity and affinity against the fungal CYP51, which makes this compound **17** a promising antifungal azole for the treatment of *C. albicans* infections [73].

In a 2015 study by Garvey et al., the efficacy of oteseconazole (**17**) was tested against *C. albicans* strains, both fluconazole-sensitive and fluconazole-resistant. In vitro and in vivo studies were performed, the latter using the murine model for vaginal candidiasis. This compound demonstrated potent inhibition of the fungal enzyme CYP51 for *C. albicans* and demonstrated adequate potency, safety profile, and adequate PK, as well as in vitro activity against resistant strains. Furthermore, it also demonstrated efficacy in the used animal model, which reinforces its promising activity as an antifungal agent [74]. In the same year, Shubitz et al. also demonstrated the efficacy of oteseconazole (**17**) for the treatment of coccidiodomycosis, by testing the compound in vitro against *Coccidioides immitis* and *Coccidioides posadasii* and, posteriorly, using the in vivo murine model. The in vitro assay revealed that oteseconazole (**17**) had high antifungal potency against the tested strains, with MIC values from 1.9 to 7.6 µM (compared to the standard drug, fluconazole, with MIC = 13–52 µM). Although the compound also showing good in vivo results, no clinical trials were performed to this date using oteseconazole (**17**) for the treatment of coccidiodomycosis [75]. It was also noted that oteseconazole (**17**) was more potent than fluconazole against most species of *Candida* and against various strains of *Cryptococcus neoformans* var. *grubii* and *C. gattii*. In the treatment of recurrent vulvovaginal candidiasis (RVVC), oteseconazole (**17**) may be an alternative to fluconazole, revealing to have high efficacy and tolerability for this condition [76].

Clinical trial results of a phase 2 study for the treatment of RVVC (NCT02267382, Table 6) were published in 2018 and revealed successful antifungal efficacy of oteseconazole (**17**). This compound demonstrated good tolerability, minimal and moderate side effects (comparable to the placebo), and recurrence rates up to 0%. In these studies, oteseconazole (**17**) also showed concordance with in vitro studies, and good PK properties, which make this compound an optimal drug candidate for the treatment of RVVC as a better alternative to fluconazole. In addition, this compound has been granted “qualified infectious disease product” (QIDP) and fast track designations from the FDA for the treatment of RVVC. Up until now, fluconazole was used, since there were no better alternatives and no approved drugs for this condition. However, oteseconazole (**17**) was approved in 2022 for the treatment of RVVC and with particular interest to women with RVVC that present resistance, allergy, or intolerance to fluconazole or even that experience drug–drug interactions due to simultaneous use of other medication [76,77,78,79].

In 2021, two clinical trial results were published: a phase 2 clinical trial for the treatment of onychomycosis of the toenail (NCT02267356, Table 6) [80] and a phase 2 clinical trial for the treatment of acute vulvovaginal candidiasis (VVC) (NCT01891331, Table 6) [81]. Onychomycosis is a chronic fungal disease which affects mostly elderly people, with limited treatment options. Adverse effects of oral drugs, limited effectiveness of topical drugs, and the risk of drug–drug interactions represent some setbacks for the arsenal of therapeutical drugs to treat this disease. Clinical trial data revealed that the oral oteseconazole (**17**) had promising antifungal activity, with complete cure rates significantly higher than the placebo and similar efficacy to that of terbinafine. Oteseconazole (**17**) is, therefore, a new treatment option with high antifungal potency and observed adequate safety and tolerability windows (which are thought to be linked to the tetrazole scaffold). Further evaluation to confirm the therapeutic effect on a larger scale should be performed [80]. Oral oteseconazole (**17**) was tested against fluconazole, the standard, for the treatment of moderate and severe VVC, a common antifungal disease in women with limited treatment due to resistance problems, drug–drug interactions, and low safety profiles. Efficacy results of oteseconazole (**17**) were similar to those of fluconazole, except for the reoccurrence of the disease, to which oteseconazole (**17**) had more successful results: no mycological recurrence was observed for this antifungal azole. Additionally, oteseconazole (**17**) presented higher safety margins and tolerability profile than fluconazole, which makes it a promising therapeutical agent for the treatment of this conditions. However, additional studies should be performed in a larger sample group to confirm the obtained data [80]. Oteseconazole (**17**) has completed six clinical trials for different conditions, which are summarized in Table 6.

Compound VT-1129, or quilseconazole (**18**, Figure 14), is a tetrazole-pyridine hybrid derived from oteseconazole (**17**). Similar to oteseconazole (**17**), this compound was studied against fungal CYP51 (in this case, using *Cryptococcus* species) and human CYP51. Not only did the compound display high affinity for the fungal enzyme, with K_d_ ranging from 14 to 25 nM (comparable to the standard drugs fluconazole, voriconazole, itraconazole, clotrimazole, and ketoconazole, with K_d_ ranging from 4 to 52 nM), but also demonstrated high selectivity for the fungal enzyme (K_d_ = 4.53 µM for human CYP51). Moreover, studies against *Cryptococcus* strains demonstrated that quilseconazole (**18**) was an effective antifungal agent, with MIC_90_ of 0.117 µM against several *C. neoformans* isolates and MIC_90_ of 0.487 µM against various *C. gatti* isolates (higher potency that fluconazole). Therefore, this compound shows promising antifungal results against this species [82].

Lockhart et al. also tested quilseconazole (**18**) antifungal activity against various *C. neoformans* and *C. gattii* isolates. Compound **18** displayed high antifungal potential, presenting MIC values from ≤0.029 to 3.9 µM and ≤0.029 to 7.8 µM for 50% and 100% of inhibition of fungal growth, respectively (lower than those of fluconazole for 50% inhibition, with MIC ranging from 0.82 to <209 µM) against *C. neoformans*. Against *C. gattii*, good antifungal potential was observed, with MIC values from ≤0.029 to 1.9 µM and 0.12 to <15.6 µM for 50% and 100% of inhibition of fungal growth, respectively. Further, the authors noted that quilseconazole (**18**) was also active against strains with reduced susceptibility to fluconazole [83].

In 2018, Wiederhold et al. assayed in vivo the antifungal efficacy of quilseconazole (**18**) for the treatment of cryptococcal meningitis, using murine models. The administration of this compound to the mice, infected with *C. neoformans*, lead to an increase survival rate (when compared to control mice) and to a reduction of colony-forming units (CFU) in the brain tissue (in comparison to control mice), which did not occur when fluconazole was administered. Although promising results against this condition, further testing needs to be performed against fungal strains with reduced susceptibility for azole antifungals [84]. Furthermore, quilseconazole (**18**) has been tested in vivo for the treatment of cryptococcal meningitis in mice, using the murine model. Results showed high efficacy in increasing survival rates and reducing the fungal burden in *C. neoformans*-infected mice. However, the use of immunocompromised mice and a susceptible fungal strain is a limitation, and further studies are necessary to further support the obtained data [85]. Quilseconazole (**18**) has completed a clinical trial and has been granted QIDP, fast track, and orphan drug designations from the FDA, for the treatment of cryptococcal meningitis [86].

**Figure 15:**
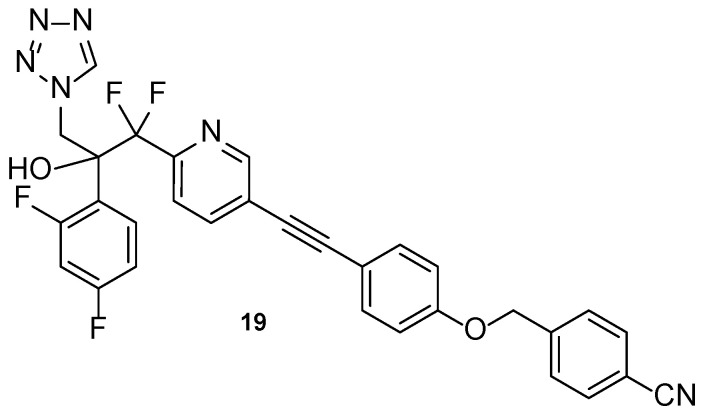
Chemical structure of VT-1598 (**19**), a next-generation tetrazole hybrid [62].

Compound VT-1598 (**19**, Figure 15) is a next generation tetrazole hybrid, with a broad spectrum against yeasts, endemic fungi, and molds, including *Candida auris* and *Aspergillus* species. It has received QIDP, fast track and orphan drug designation by the FDA for the treatment of coccidioidomycosis. Additionally, in vivo studies using the murine model have demonstrated the antifungal activity of VT-1598 against invasive aspergillosis and are an important base for future studies [62,87]. Information about clinical trials of this compound is presented in Table 6.

### 4.2. New Molecules with a Traditional Azole Pharmacophore

#### 4.2.1. New Triazoles

**Figure 16:**
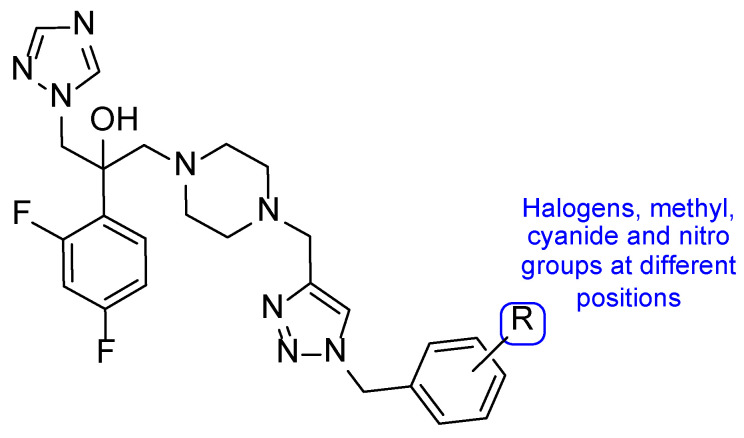
Chemical structure of the tested triazole compounds [96].

Inspired in 2nd generation antifungal azoles, Wang et al. designed novel triazole molecules, combining the triazole ring with a hydroxyl substituent, a difluorophenyl group and a piperazine containing side chain (Figure 16). The compounds’ antifungal activity was tested against two strains of *C. albicans*, *C. parapsilosis*, *C. neoformans*, *C. glabrata*, *A. fumigatus*, *T. rubrum*, and *N. gypsea* using the broth microdilution method. The therapeutical antifungal drugs itraconazole, fluconazole, and voriconazole were used as standards, and results were presented as MIC_80_. Generally, the tested compounds were not effective against *C. glabrata* (MIC_80_ values from 8 to >126 µM) and *A. fumigatus* (MIC_80_ > 126 µM), presented moderate activity against *T. rubrum* (MIC_80_ from 1.7 to 30.8 µM), good to moderate activity against *N. gypsea*, *C. parapsilosis*, and *C. neoformans* (MIC_80_ values ranging from 0.5 to 31.2 µM) and showed good to high antifungal activity against both strains of *C. albicans* (MIC_80_ from 0.44 to 30.8 µM, with the exception of one compound). To sum, some of the tested compounds displayed promising antifungal potential and could be further evaluated [96].

**Figure 17:**
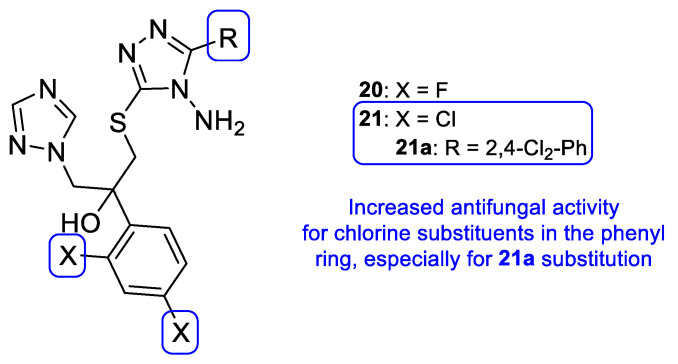
Scaffold for the difluoro- (**20**) and dichloro- (**21**) phenylethyl-triazole series, as well as the structure for compound **21a** and respective SAR [97].

New triazole compounds were developed by replacing the 1,2,4-triazol-1-yl group in fluconazole with a 4-amino-3-mercapto-1,2,4-triazole moiety (Figure 17). This series of compounds was tested using the broth microdilution method against four strains of *C. albicans*, *C. parapsilosis*, *C. neoformans*, *E. floccosum*, and *T. mentagrophytes*, using fluconazole as standard. Compound **21a** (Figure 17) presented the highest antifungal potential, with MIC values ranging from 3.8 to 120 µM against all tested fungi (fluconazole with MIC values ranging from 0.82 to 52.2 µM for all tested strains, except for *T. mentagrophytes*, to which it was inactive in this study). Additionally, the authors concluded that the dichlorophenylethyl-triazole structure (**21**) is preferred for antifungal activity over the difluoro- (**20**) [97].

**Figure 18:**
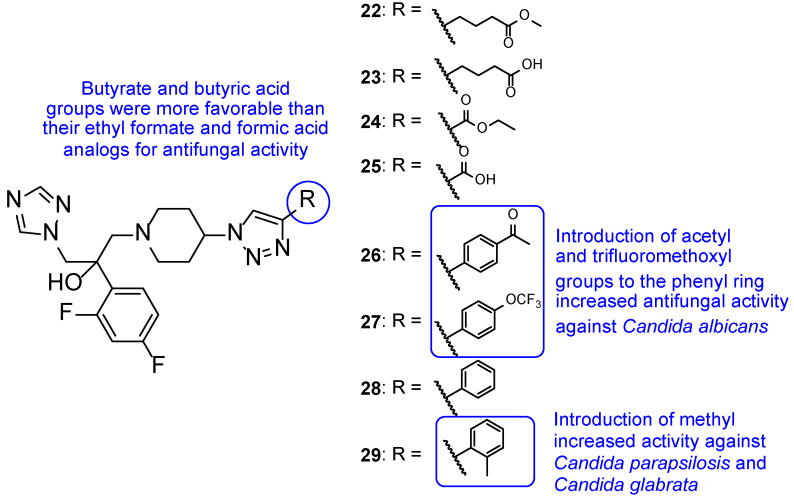
Chemical structures for compounds **22**–**29**, and respective SAR [98].

Later in 2014, a new series of triazole-piperidine compounds was designed, synthesized, and tested for antifungal activity against a variety of fungi (*C. albicans*, *C. neoformans*, *C. parapsilosis*, *C. glabrata*, *A. fumigatus*, *T. rubrum*, and *N. gypsea*) and using fluconazole and itraconazole as standards. Generally, the tested piperidine-containing triazoles **22**–**29** (Figure 18) displayed good inhibitory activity against all tested strains, in particular for *C. albicans* and *C. neoformans*. Given the obtained results, the authors suggested that compounds **22** and **23**, with butyrate and butyric acid, respectively, presented higher antifungal potential than their analogs **24** and **25**, with ethyl formate and formic acid groups, respectively. Moreover, the introduction of 4-acetyl (**26**) and 4-trifluoromethoxyl (**27**) to the phenyl ring of compound **28** resulted in increased antifungal activity against *C. albicans* and introduction of 2-methyl (**29**) resulted in increased activity against *C. parapsilosis* and *C. glabrata* (Figure 18). Compounds with aromatic rings presented excellent antifungal activity and compounds **26**, **27**, and **29** were considered the most active compounds, and, therefore, promising antifungal leads [98].

**Figure 19:**
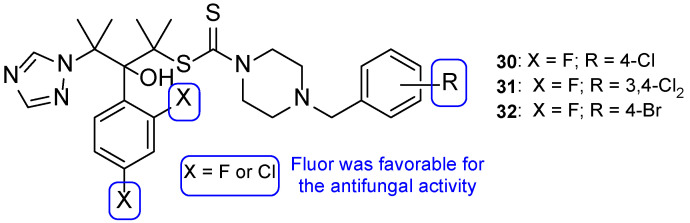
Chemical structures for compounds **30**–**32** and SAR for the phenyl ring [99].

To enhance some properties of fluconazole, Mahmoudi et al. designed fluconazole derived triazole alcohols, with a *N*-(halobenzyl)piperazine carbothioate side chain. The new compounds were tested for their antifungal activity against *C. albicans*, *C. glabrata*, *C. parapsilosis*, *C. krusei*, and *C. tropicalis* using the broth microdilution method. All tested compounds presented great results against all tested strains, with MIC values comparable or better than those of fluconazole. Compound **30** (Figure 19) displayed excellent results (MIC values ranging from 0.11 to 0.86 µM against all tested fungi) when compared to fluconazole (MIC values ranging from 1.6 to 13.1 µM). Against fluconazole-resistant strains, **30** also showed better results than fluconazole (MIC values from 3.4 to 27.6 µM, while fluconazole was inactive) and compounds **31** and **32** (Figure 19) were also potent against all resistant strains (MIC values ranging from 0.4 to 52 µM). Moreover, the authors concluded that compounds substituted with two fluor substituents at C-2 and C-4 of the phenyl ring were more favorable than their chlorine analogs (Figure 19). Since compound **30** presented excellent results, it was also tested for toxicity against HepG2 cells and erythrocytes, where it displayed minimal toxicity and favorable safety profile, revealing a promising structure for antifungal activity [99].

**Figure 20:**
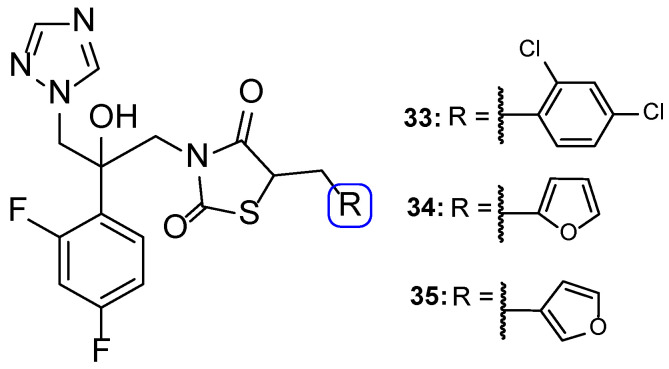
Chemical structures of triazole-thiazolidinedione hybrid compounds **33**–**35** [100].

In 2014, Wu et al. designed and synthesized a series of new fluconazole analogs, utilizing a thiazolidinedione scaffold instead of one of its triazole rings. The antifungal effects of the compounds were assessed using the broth microdilution method as values of MIC_80_ and fluconazole was used as standard. Compounds **33**–**35** (Figure 20) presented good inhibitory activity against all tested strains (*C. albicans*, *C. parapsilosis*, *C. neoformans*, *C. glabrata*, *C. tropicalis*, *A. fumigatus*, *T. rubrum*, and *N. gypsea*) and demonstrating particularly high effect against *C. albicans*, with MIC_80_ values of 0.030, 0.15, and 0.15 µM, respectively (MIC_80_ of fluconazole was 0.81 µM). Additionally, **34** and **35** presented effective values against four fluconazole-resistant isolates of *C. albicans*, with MIC_80_ values ranging approximately from 18.50 to 148.01 µM for compound **34** and 37.00 to 74.00 µM for compound **35** (MIC_80_ values for fluconazole were >3343.35 µM for all tested isolates). These values show that these triazole-thiazolidinedione hybrids are promising antifungal leads [100].

**Figure 21:**
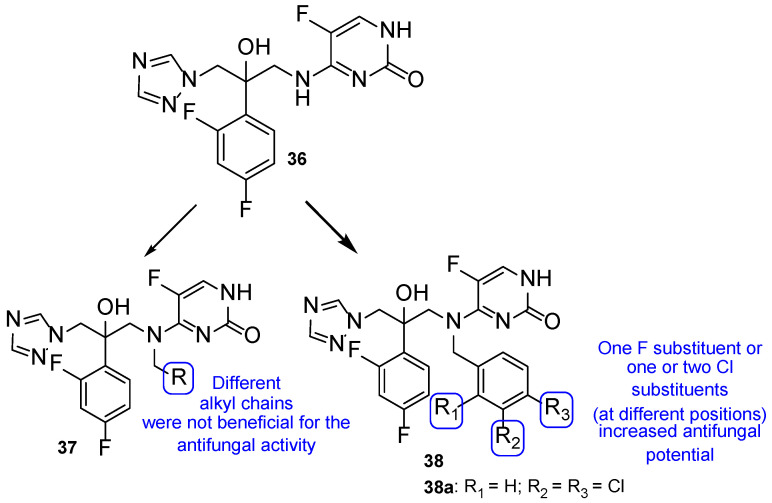
Chemical structure of 5-flucytosine and fluconazole hybrid (**36**) and of scaffolds of aryl series (**37**) and halobenzyl series (**38**), and particularly of compound **38a**, and SAR for the two series [101].

The antimycotic 5-flucytosine is an agent utilized therapeutically in combination with other antifungal drugs, including azoles, due to its narrow spectrum, weak antifungal potential, and fast appearance of resistance mechanisms. As fluconazole has several good proprieties, such as good oral bioavailability and absorption, strong antifungal activity, broad spectrum, safety profile, and adequate PK, Fang et al. designed and synthesized new 5-flucytosine-fluconazole hybrids. Given that this combination of azoles with 5-flucytosine presented great antifungal activities, the authors decided to come up with a new series of hybrids, using the two different molecular scaffolds of 5-flucytosine and fluconazole, and study their antifungal potential. The main goal of this study was to exploit a new series of potential antifungal drugs, obtained to hopefully increase the spectrum of action and avoid resistance problems and adverse effects [101]. The synthesized compounds were tested for antifungal activity against *C. albicans*, *C. parapsilosis*, *A. fumigatus*, and *C. tropicalis*, using the broth microdilution method and 5-flucytosine and fluconazole as standards. The hybrid **36** (Figure 21) presented moderate to high antifungal potential (with MIC values ranging from 40 to 170 µM) when compared to the drugs used as positive controls, fluconazole and 5-flucytosine (with MIC values ranging from 3 to 1650 µM and 30 to 1980 µM, respectively). These values suggest that the hybridization was successful, meaning it favored the antifungal activity [101].

Thenceforth, structural modifications were made to compound **36** (mainly introduction of alkyl and aryl groups) to illustrate SAR. Two new series of derivatives **37** and **38** were synthesized, with the scaffold presented in Figure 21. The **37** series presented weak to no antifungal activities, which led to the conclusion that the introduction of alkyl substituents at that site was not beneficial for the activity. On the other hand, halobenzyl compounds (**38**) showed lower values of MIC in comparison to the aryl series (**37**), which suggested that this structural modification was useful in order to increase the antifungal potential. In particular, compound **38a** with a 3,4-dichlorobenzyl substituent presented the best MIC values, ranging from 9 to 30 µM. In addition, **38a** also presented good lipophilicity and rapid fungicidal potential (assessed against *C. albicans* via time-kill kinetic assay), thus being a promising scaffold for a new antifungal drug. In addition, docking, quantum chemical, and computational studies were performed to assess the mechanism of action, binding mode, and PK properties, which will not be discussed herein [101].

**Figure 22:**
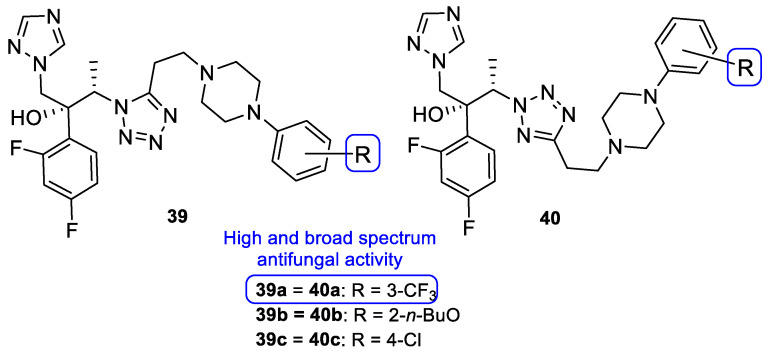
Chemical structures of compounds **39a**–**c** and **40a**–**c** and SAR [102].

Upadhayaya et al. synthesized a variety of tetrazole-1,2,4-triazole hybrids and their respective positional isomers (Figure 22) in order to study their in vitro antifungal activity against *C. albicans*, *C. tropicalis*, *C. parapsilosis*, *C. krusei*, *C. glabrata*, *C. neoformans*, *A. fumigatus*, and *Aspergillus niger* [102]. The method used for this study was the broth microdilution method, and the results for the antifungal activity were presented in MIC. The tetrazole-triazole hybrid **39a**, presenting a 3-trifluoromethyl on the phenyl ring of piperazine showed broad and strong antifungal activity, with MIC values ranging from 0.21 to 1.73 µM (when compared to the standard drugs fluconazole and itraconazole, with MIC values ranging from 0.4 to >26 µM and 0.01 to 0.7 µM, respectively, not concordant with literature results for fluconazole for *A. fumigatus*, to which it is normally inactive). The positional isomer of compound **39a**, **40a**, presented similar antifungal activity, with MIC values ranging from 0.43 to 1.73 µM. Both these positional isomers were more active than fluconazole against resistant fungal *Candida* species. Compounds **39b** and **40b**, which presented a 2-butoxy substituent on the phenyl ring also presented interesting activity against all tested strains, with MIC values ranging from 0.43 to 1.72 µM and 0.43 to 3.44 µM, respectively (with the exception for both *Aspergillus* strains with values >13.8 µM values). By analyzing the results, it was concluded that these compounds behaved similarly to the control drug itraconazole and with better MIC values than fluconazole against *Candida* strains. Compound **39c**, with a 4-chloro substituent on the phenyl ring of piperazine, also presented good MIC values (ranging from 0.92 to 3.68 µM) against tested *Candida* strains. Its positional isomer, **40c**, showed similar results. Other substituents in R presented moderate activity (which was the case for 4-nitrophenyl and 4-fluorophenyl), while some showed moderate to weak antifungal activity (such as 2-methoxyphenyl and 3-chlorophenyl). The configuration of the synthesized hybrids (*2R,3S*) was also important since it enhanced the activity when compared to the non-optically active congeners. Given the data presented, compounds **39a** and **40a**, with a trifluoromethyl group at C-3, were the most promising of the synthesized hybrids, displaying broad spectrum of action and low values of MIC [102].

#### 4.2.2. Tetrazoles

**Figure 23:**
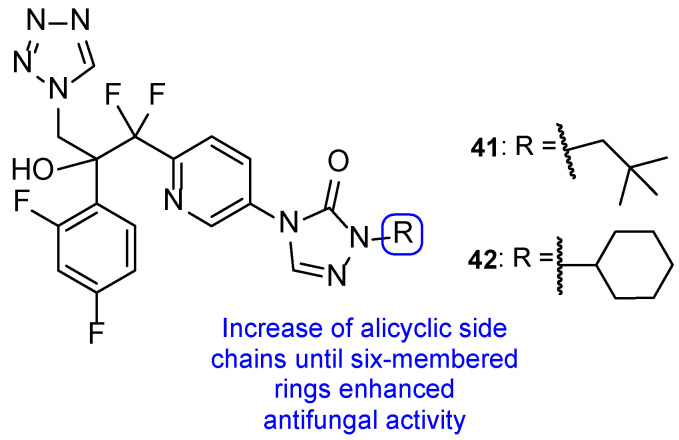
Chemical structures of compounds **41** and **42**, and SAR [103].

In 2018, Qian and co-workers hybridized a tetrazole moiety with a 4-pyridinyl-1,2,4-triazole-3-one moiety, resulting in a series of new compounds (Figure 23) [103]. The authors synthesized various sets of compounds with different substitutions on the triazolone ring, namely on the C-2 position, and studied their antifungal activities against two strains of *C. albicans*, *C. parapsilosis*, *C. glabrata*, *C. neoformans*, and *A. fumigatus*. Firstly, the substituents were different hydroxyl chains, all of which proved to be inactive. Subsequently, those side chains were replaced with aliphatic side chains, to improve the lipophilicity of the hybrids. The substitution proved to be successful and resulted in a series of compounds with good MIC_80_ values against *Candida* species and *C. neoformans* (which were superior when compared to the standards fluconazole and VT-1161). From those compounds, **41** presented the best MIC_80_ values, ranging from 0.49 to 1.97 µM (except for *A. fumigatus*). Following that, the authors concluded that the antifungal potential was enhanced with the volume increase of those side chains and with lipophilicity increase. Additionally, it was also noted that the lipophilicity ameliorated the compounds capacity to penetrate the fungal membrane and the added volume would better fit the fungal CYP51 hydrophobic pocket—both verified by supplementary docking studies. Considering these findings, Qian et al. developed compounds with aromatic moieties with benzyl and pyridyl substituents, which presented inferior antifungal values of MIC_80_ to those of compound **41**. Bearing in mind previous results, compounds were synthesized with the introduction of lipophilic and bulky alicyclic side chains. The antifungal potential of these compounds increased continuously with the aliphatic ring size, until no further than six-membered rings. Aliphatic rings with seven or eight carbon atoms revealed to decrease the activity in comparison to smaller rings. Once again, these results were concomitant with the statement that the antifungal potential was increased with suitable lipophilicity and volume of the side chain. From this series of compounds synthesized, it is important to highlight compound **42**, which exhibited excellent MIC_80_ values ranging from 0.24 to 0.48 µM (except for *A. fumigatus*), when compared to compound **41** and the standard drugs. Additionally, **41** and **42** presented good results against *N. gypsea* (with MIC_80_ values of 7.9 and 7.7 µM, respectively), which was resistant to the reference drug VT-1161, and weak inhibition against human CYP450s, which makes these promising compounds for further studies with good selectivity and low probability of drug–drug interactions [103].

Up to now, several azole antifungal compounds have been presented in this review. Amongst these derivatives, a common pharmacophoric structure can be envisioned (Table 7, entry 1). Many azole agents in development contain this main structure, such as isavuconazole (**3**), iodiconazole (**4**), and the tetrazole-pyridine hybrids (VT-1161, VT-1129 and VT-1598), with different substituents at R and triazole or tetrazole rings at X. From various SAR studies and by analyzing the results of different studies with a large arsenal of compounds, a compilation of enhancing features for the antifungal activity has been summarized (Table 7). In some cases, chirality issues were taken into consideration since enantiomers vs. racemates can produce different spectra of activity. In other cases, those studies were not taken into account and further testing should be performed to assess the impact of chirality.

### 4.3. Novel Derivatives with Azole Moieties Aside the Traditional Pharmacophore

**Figure 24:**
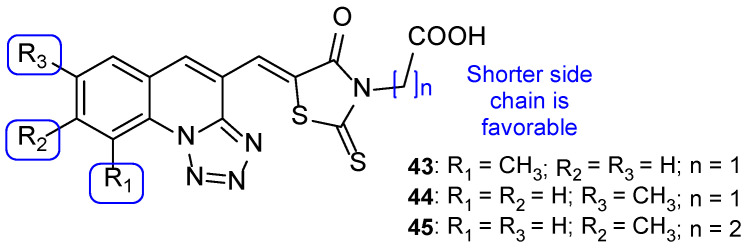
Chemical structures of **43**–**45** and SAR [104].

Apart from this common scaffold, Subhedar et al. synthesized novel tetrazole-quinoline hybrids, containing rhodanine, and studied them for their antifungal activity against *C. albicans*, *Aspergillus flavus*, *A. niger*, *C. neoformans*, and *Fusarium oxysporum*. The compounds generally presented weak to moderate antifungal activity, with MIC values ranging from 76 to 454 µM against all tested strains. Compounds **43**, **44**, and **45** (Figure 24) where the most promising ones, with MIC values similar to that of the standard drug, miconazole, against *C. albicans* (MIC values of 65, 65, 63, and 60 µM, respectively). SAR studies demonstrated that a shorter side chain (n = 1) was preferable than a longer one (n = 2) in terms of the antifungal potential; also, the introduction of a carboxylic acid increased antifungal activity against *F. oxysporum*, *A. flavus*, and *C. albicans* (when compared to the unsubstituted analogs) (Figure 24). Additionally, in silico absorption, distribution, metabolism, and excretion (ADME) prediction showed these hybrids present good ADME properties [104].

**Figure 25:**
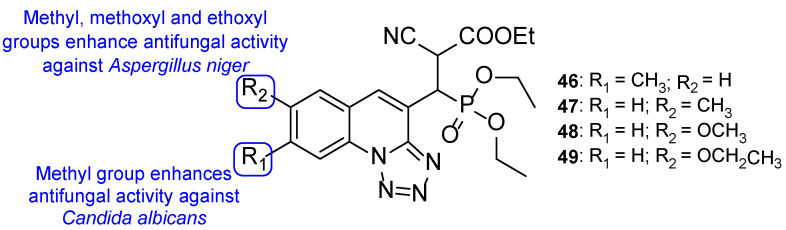
Chemical structures for compounds **46**–**49** and SAR [105].

Regarding the previous compounds, new hybrids were synthesized by Kategaonkar et al. by replacing the rhodanine fragment with an ethoxy phosphonyl group [105]. These compounds were tested against *C. albicans* and *A. niger* and demonstrated moderate antifungal activity. Compound **46** (Figure 25) showed comparable activity to that of griseofulvin, the standard drug (MIC value of 22 µM vs. 28 µM for griseofulvin) against *C. albicans*. Compounds **47**, **48**, and **49** (Figure 25) also presented similar antifungal activity as griseofulvin, with MIC value of 22, 22, and 21 µM, respectively (griseofulvin value of MIC was 28 µM) against *A. niger*. These results suggest that the presence of a methyl group at C-7 in **46** results in increased antifungal activity against *C. albicans* and the presence of methyl, methoxyl, and ethoxyl groups at C-8 (**47**, **48**, and **49**, respectively) enhanced antifungal potency against *A. niger* [105].

**Figure 26:**
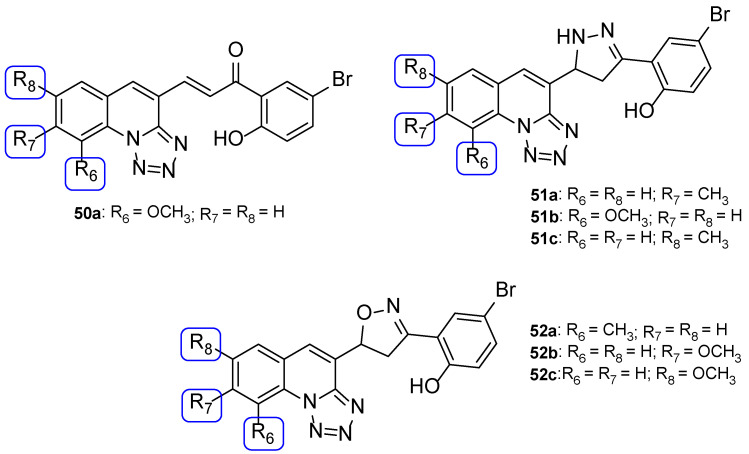
Chemical structures for compounds **50a**, **51a**–**c**, and **52a**–**c** [106].

Nikam et al. synthesized chalcone, pyrazoline, and isoxazoline hybrids with tetrazole moieties and tested their antifungal activity against *C. albicans* and *A. niger* using the agar dilution method and fluconazole as standard. All tested compounds displayed moderate antifungal activity when compared to fluconazole (MIC values of 82 µM against both strains). Compounds **50a**, **51a**, **51b**, **52a**, **52b**, and **52c** (Figure 26) presented similar values of MIC to the reference drug (MIC = 82 µM) against *C. albicans* and compounds **50a**, **51c**, and **52c** (Figure 26) displayed equal values of MIC to standard (MIC = 82 µM) against *A. niger*, making them promising structures for antifungal activity [106].

**Figure 27:**
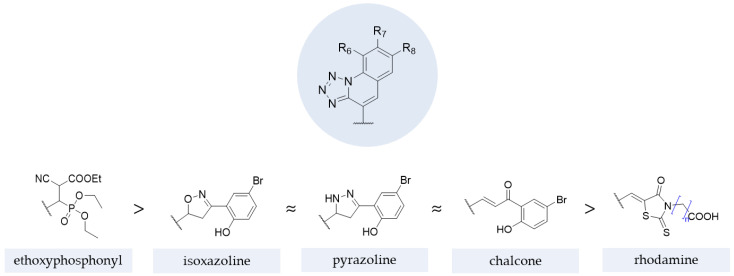
Impact of different moieties on the antifungal activity of the represented scaffold [104,105,106,107].

The results from studies with these structures demonstrated that, generally, the contribution for antifungal activity against *C. albicans* and *A. niger* was higher with ethoxyphosphonyl moieties (tested by Kategaonkar et al. [105]), approximately the same for oxazoline, pyrazoline, and rhodanine moieties (tested by Nikam et al. [106] and Subhedar et al. [104]) and, lastly, for the chalcone moiety (tested by Nikam et al. [106]). The SAR extracted from these structures and substructures is represented in Figure 27.

**Figure 28:**
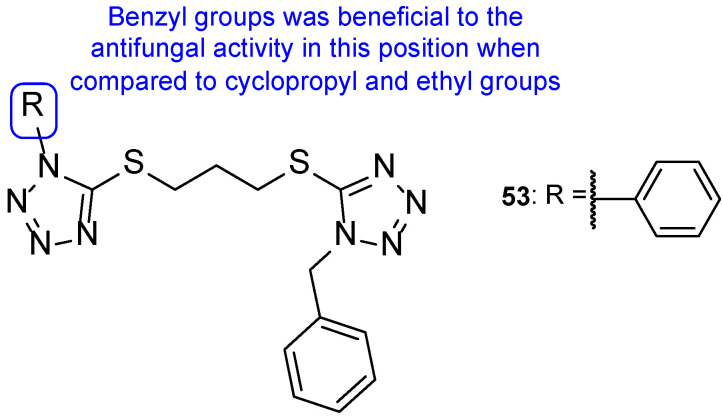
Chemical structure of compound **53** and SAR for this series of tetrazole-tetrazole hybrids [108].

In 2011, a group of researchers decided to synthesize a series of tetrazole hybrids with a thiol substituent. To study their antifungal potential, compounds were tested against *A. fumigatus*, *A. flavus*, *T. mentagrophytes*, and *Talaromyces marneffei* and their MIC were measured. Compound **53** (Figure 28) presented high antifungal activity against all tested fungal strains, with MIC values ranging from 44 to 49 mM, comparable values to the standard itraconazole (MIC values ranging from 27 to 30 mM). When compared to other hybrids in this series of compounds, the benzyl substituent (**53**) was beneficial instead of the cyclopropyl and ethyl groups in the same position [108].

**Figure 29:**
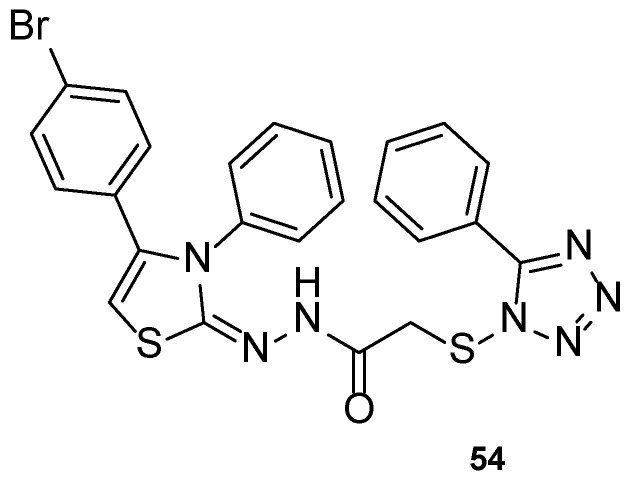
Chemical structure of compound **54** [109].

Altıntop et al. developed a new series of tetrazole-thiazoline hybrids and tested the antifungal activity against Aspergillus parasiticus, Aspergillus ochraceus, Penicillium chrysogenum, Trichoderma harzianum, Fusarium solani, Fusarium moniliforme, Fusarium culmorum, and C. albicans. All compounds displayed good antifungal activity against all tested strains, with MIC values similar or higher than the standard, ketoconazole (with the exception of P. chrysogenum). Particularly, compound **54** (Figure 29) showed higher antifungal activity than ketoconazole against C. albicans, with MIC value of 221 µM (compared to MIC value of 470 µM for the standard drug). In this study, the authors concluded that compound **54** was the most promising synthesized hybrid, due to its antifungal results. Additionally, MTT assay results highlighted its low toxicity against NIH3T3 cells (IC_50_ = 433 ± 28.9 µg/mL) [109].

**Figure 30:**
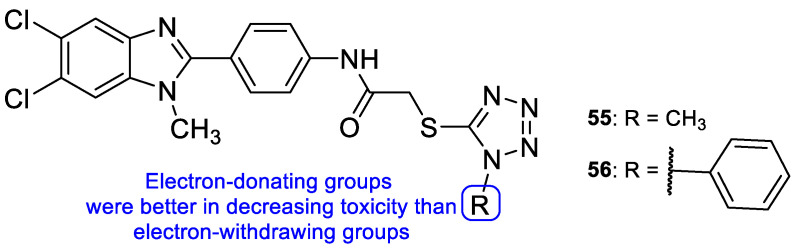
Chemical structures of compounds **55** and **56**, and toxicity SAR [110].

In 2011, a series of tetrazole-benzimidazole compounds was synthesized and tested for its antimicrobial activity. Benzimidazole, which is considered an interesting scaffold and is present in various compounds with effective antimicrobial properties (including antifungal), was utilized in combination with other azoles, in search for new antifungal drugs. The antifungal activity (MIC) was evaluated against *C. albicans*, *C. glabrata*, and *C. tropicalis*. Compounds **55** and **56** (Figure 30) presented high antifungal activity, with MIC values of 56 and 49 µM, respectively, to *C. albicans* and *C. glabrata* and 112 and 98 µM, respectively, to *C. tropicalis* (when compared to the standard drug, fluconazole, which presented values of 163, 82 and 163 µM to *C. albicans*, *C. glabrata*, and *C. tropicalis*, respectively). However, compound **56** with a 1-phenyl-1*H*-tetrazol-5yl-thio substituent was demonstrated to be toxic using the brine-shrimp toxicity assay, assumed to be linked to its lipophilicity. Moreover, it was noted by the authors that the presence of *N*-methyl groups, which are electron-donating, in the azole rings (**55**) resulted in a decreased toxicity in comparison to compounds with electron-withdrawing groups (**56**) [110].

**Figure 31:**
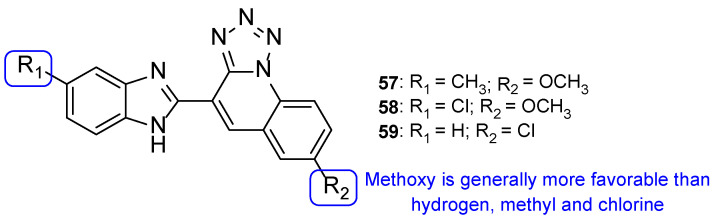
Chemical structures of compounds **57**–**59** and SAR regarding the R_2_ substituents [111].

In the same year, Mungra and co-workers also produced tetrazole-benzimidazole hybrids and studied their antifungal activity against two fungal strains, *A. fumigatus* and *C. albicans*, using the broth microdilution method. All tested compounds exhibited weak antifungal activity against *A. fumigatus*. Compounds **57**, **58**, and **59** (Figure 31) presented promising activity against *C. albicans*, with MIC values of 757, 713, and 390 µM, respectively, when compared to the standard griseofulvin, with MIC = 1417 µM. Additionally, the authors concluded that methoxyl substituents at R_2_ led to stronger activity than hydrogen, methyl, or chlorine substituents [111].

**Figure 32:**
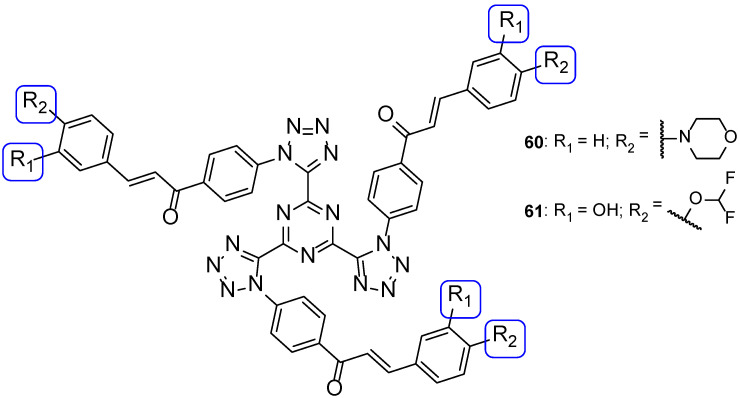
Chemical structures of **60** and **61** [112].

In another study, tri-tetrazoles compounds were synthesized and tested in vitro for their antifungal properties, by measuring the MIC against *C. albicans*, *Saccharomyces cerevisiae*, *A. niger*, and *A. fumigatus*. The most promising compounds out of the series synthesized were **60** and **61** (Figure 32), which displayed good antifungal properties against all tested strains (with MIC values ranging from <0.14 to 1.08 and <0.14 to 0.54 µM, respectively, comparable to the standard drug fluconazole, with MIC values ranging from <0.52 to 1.02 µM, not concordant with literature reports for *A. fumigatus*, to which it is normally inactive). Due to the observed MIC values and the broad spectrum, these were considered promising leads for antifungal therapy. The presence of a tetrahydro-1,4-oxazine, morpholine, in compound **60** and the presence of a hydroxyl and difluoro methoxyl groups on the phenyl ring in compound **61**, associated with good antifungal activities, suggested that these groups in these positions may increase the antifungal potential [112].

**Figure 33:**
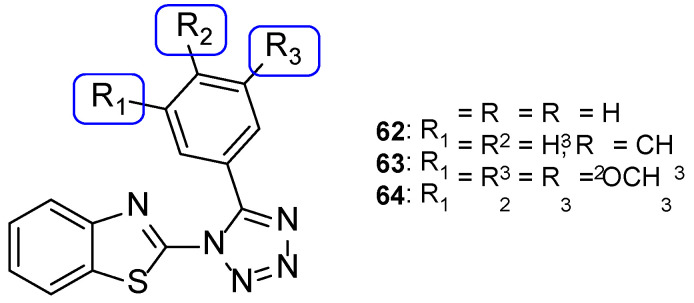
Chemical structures of compounds **62**–**64** [113].

Benzothiazole-tetrazole hybrids were synthesized by Shanmugapandiyan and Atmakuru to assay their antifungal activity against two fungal strains, *A. niger* and *C. albicans*, using the paper disc diffusion method. All tested compounds showed significant antifungal potential against both fungal strains. Compounds **62**, **63**, and **64** (Figure 33) exhibited high inhibition zone diameter (DIZ) values for both species (21, 20, and 21 mm, respectively, against *A. niger*, and 23 mm against *C. albicans* at the concentration of 250 µg/mL), although they were smaller than the standard fluconazole (values of 22 and 25 mm against *A. niger* and *C. albicans*, respectively, at the concentration of 250 µg/mL) [113].

**Figure 34:**
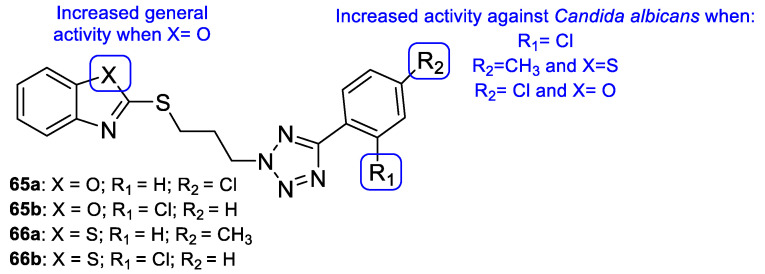
Chemical structures of compounds **65a**–**b** and **66a**–**b**, and SAR [114].

Łukowska-Chojnacka et al. tested newly synthesized compounds with tetrazole and benzothiazole/benzoxazole moieties against *C. albicans*, *A. niger*, *Colletotrichum coccodes*, *Fusarium sambucinum*, and *F. oxysporum*, to determine their antifungal activities. All compounds presented little to no activity against all strains, except for *C. albicans*. Despite the disappointing results, the authors were able to deduce that benzoxazole hybrids **65** presented generally better antifungal activity than the corresponding benzothiazoles **66** (with percentage of growth inhibitions ranging from 0 to 36.7 vs. 0 to 46.7) against the mold species (Figure 34). In the case of *C. albicans*, compounds **65a**, **65b**, **66a**, and **66b** (Figure 34) showed high antifungal activity (with percentages of cell inhibition from 98.80% to 99.36% at the lowest tested concentration, 0.0313 µg/mL) [114].

**Figure 35:**
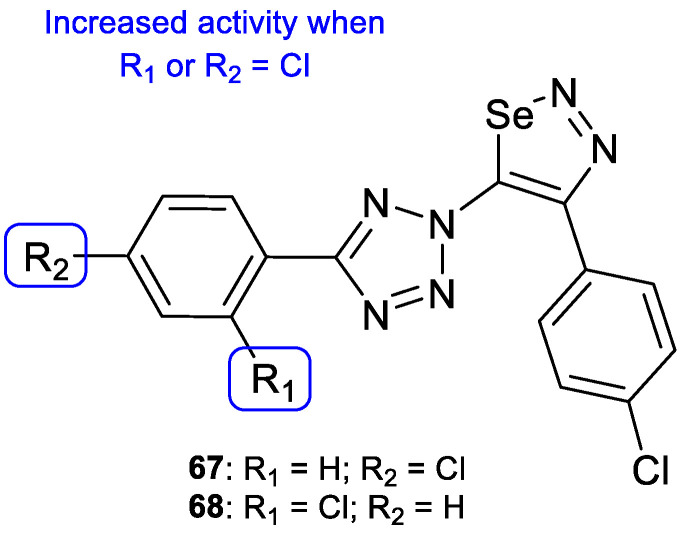
Chemical structures of compounds **67** and **68** and SAR against *C. albicans* and *A. niger* [115].

Selenadiazoles are heterocyclic compounds which present various biological activities, such as antibacterial, antifungal, antitumor, among others. The addition of this moiety to known antifungal azoles, such as tetrazole, may in some cases lead to an increased activity. In 2015, Kanakaraju and Suresh designed a new series of hybrids using tetrazole and selenadiazoles and evaluated the antifungal activity using the broth microdilution method against *C. albicans* and *A. niger*. All tested compounds, with the exception of **67** and **68** (Figure 35), presented only weak antifungal activity against both strains. Compound **67** showed promising values when compared to fluconazole against *A. niger* (MIC = 14.8 µM vs. 20.4 µM) and **68** presented promising values of MIC against *C. albicans* (MIC = 14.8 µM vs. 20.4 µM). In the presence of the selenadiazole ring (when compared to compounds without it), there was an increased antifungal activity, in particular when the chlorine substituent was present on the phenyl ring linked to tetrazole (Figure 35) [115].

**Figure 36:**
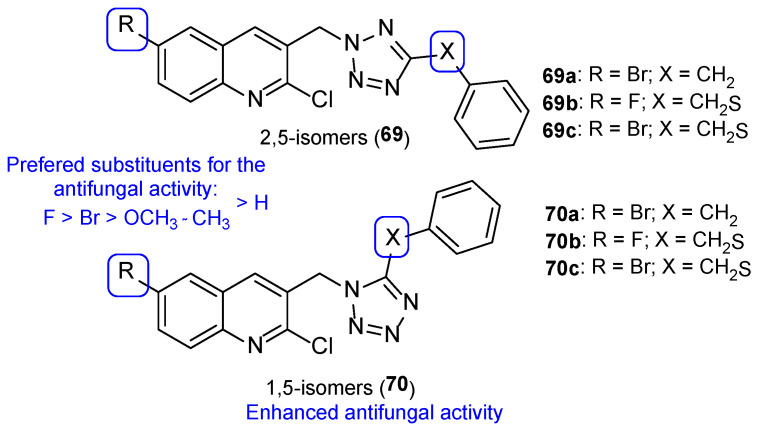
Chemical structures of 2,5-isomers **69a**–**c** and 1,5-isomers **70a**–**c** and SAR [116].

Later, Shaik et al. synthesized tetrazole-quinoline hybrids (2,5 (**69**) and 1,5-regioisomers (**70**)) which were assayed for antifungal activity using the disc diffusion method against *C. albicans* and *A. fumigatus* and using fluconazole as standard. Generally, the tested compounds presented good antifungal activity for both strains, with MIC values ranging from 24 to 130 µM against *C. albicans* and 6.5 to 149 µM against *A. fumigatus* (fluconazole presented MIC = 98 µM for both strains, which is not in concordance with literature reports—fluconazole is usually inactive to *A. fumigatus*). Compound **69a** and **70a** (Figure 36), with a bromine substituent at C-6, displayed good antifungal activity against both strains, with MIC = 24 µM. Moreover, compounds **69b** and **70b** (Figure 36), with a fluor substituent at C-6 and **69c** and **70c** (Figure 36), with a bromine substituent at C-6, showed great antifungal activity against *A. fumigatus*, with MIC values of 6.5, 6.5, 11.2, and 11.2 µM, respectively. The obtained results not only suggested that the 1,5-regioisomer presented better values of fungal inhibition, which reveals regioselectivity of the enzyme, but also that compounds with halogen groups (such as fluorine and bromine) at the C-6 led to an increase in activity (other groups, such as methyl, methoxyl, and hydrogen groups, were less contributive). SAR with the order of preference for substituent at C-6 is represented in Figure 36 [116].

**Figure 37:**
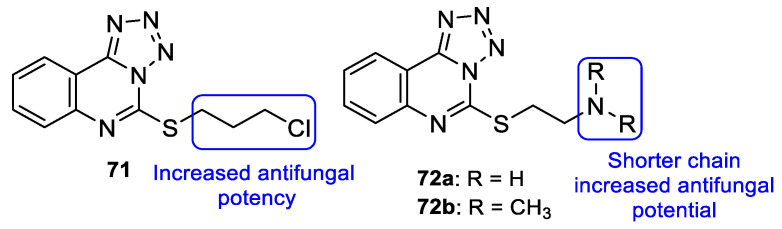
Chemical structures of compounds **71** and **72a**–**b,** and SAR [117].

In 2013, Antypenko et al. synthesized a series of compounds and tested them for their antifungal activity against *C. albicans*. Compound **71** (with a chloropropyl substituent) (Figure 37) was the most potent compound, with DIZ of 22 mm at the concentration of 100 µg/disk (when compared to nystatin, the standard, with DIZ of 21 mm at the concentration of 100 µg/disk). Moreover, in compounds **72a** and **72b** (Figure 37), the authors observed that the shortening of the dialkylamino fragment resulted in an increase of the activity [117].

**Figure 38:**
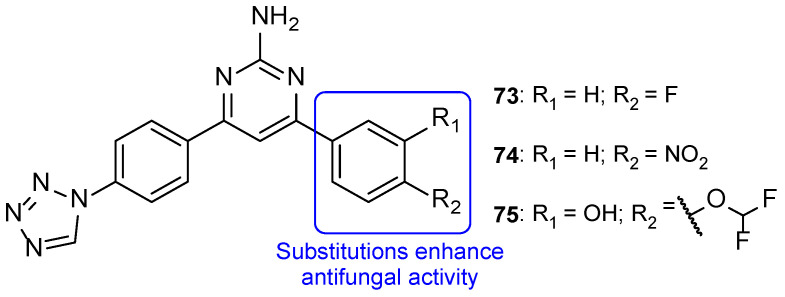
Chemical structures of compounds **73**–**75**, and SAR regarding the phenyl ring [118].

The series of tetrazole-pyrimidine synthesized by Vembu et al., tested using the broth microdilution method against *C. albicans*, *S. cerevisiae*, *A. niger*, and *A. fumigatus*, showed great potency against the tested fungi (MIC < 0.4 to 15 µM, apart from one value) compared to MIC values of fluconazole (MIC ranging from 0.52 to 8.2 µM—not concordant with literature results for *A. fumigatus*). Compounds **73**, **74**, and **75** (Figure 38) demonstrated high antifungal activity against all strains (with MIC values ranging from <0.4 to 15 µM), with **75** being the most effective one and, in some cases, stronger than fluconazole. Additionally, it was noted that compounds with substitutions on the phenyl ring are generally more active than the unsubstituted ones [118].

**Figure 39:**
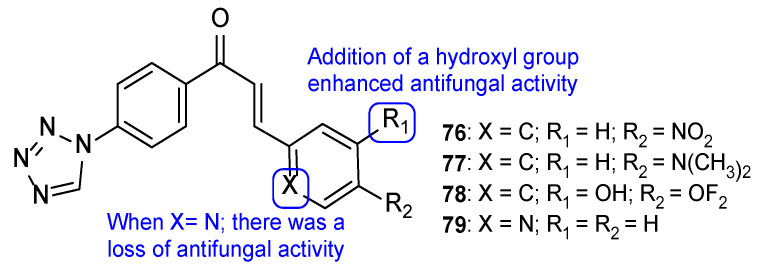
Chemical structures for compounds **76**–**79**, and SAR [119].

A series of tetrazole-chalcone hybrids was synthesized by Vembu et al. and evaluated for its antifungal activity against *C. albicans*, *A. niger*, and *A. fumigatus* and using fluconazole as standard. All tested compounds displayed antifungal activity against the tested strains (with MIC values ranging from <0.46 to 18 µM). Compounds **76** and **77** (Figure 39) demonstrated good MIC values, ranging from <0.5 to 0.98 µM, and compound **78** (Figure 39) showed the best antifungal potential of the series, with MIC values ≤ 0.46 µM against all tested strains (when compared to fluconazole, which presented MIC values from <0.52 to 8.2 µM, not concordant with literature results for *A. fumigatus*). The addition of a nitrogen atom on the aromatic ring, leading to a pyridinyl ring (compound **79**), resulted in a loss of antifungal activity, and introduction of a hydroxyl group (compound **78**) led to enhanced antifungal potential (Figure 39) [119].

**Figure 40:**
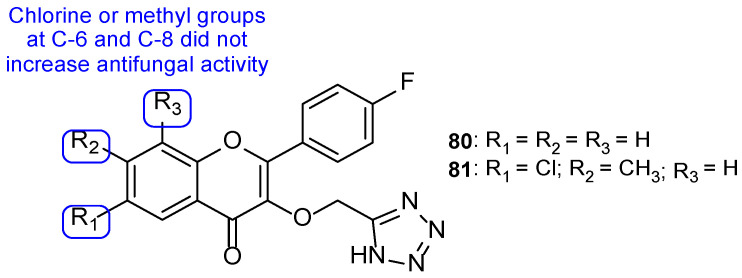
Chemical structures for compounds **80** and **81**, and SAR regarding the phenyl ring [120].

Dofe et al. synthesized a novel series of tetrazole-flavone compounds and assayed for antifungal activity using the broth microdilution method against *C. albicans*, *C. glabrata*, and *C. tropicalis*. All tested compounds presented higher antifungal activity than the standard miconazole against *C. glabrata* and *C. tropicalis*. When tested against *C. albicans*, compounds **80** and **81** (Figure 40) showed promising antifungal activity, comparable to that of miconazole (MIC = 32.6, 32.3, and 30 µM, respectively). It was noted that substitutions in C-6 and C-8 on the phenyl ring with methyl and chlorine did not increase the antifungal activity, when compared to the unsubstituted ring (compound **80**) (Figure 40). In addition, all tested compounds inhibited biofilm formation [120].

**Figure 41:**
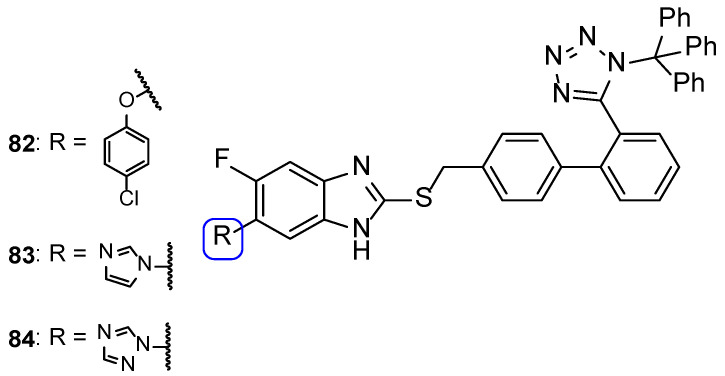
Chemical structures of compounds **82**–**84** [121].

A new series of hybrids was synthesized and tested for its antifungal activities by Nandha et al. against *C. albicans*, *C. glabrata*, *C. krusei*, and *C. tropicalis* using the broth microdilution method. The compounds showed little to moderate antifungal activity, with MIC values ranging from 5.6 to > 89.7 µM (when compared to the reference drug fluconazole, with MIC values ranging from 3.7 to 13.1 µM). Compounds **82**, **83**, and **84** (Figure 41) were the most promising ones, which suggested that these substituents could lead to an increase of antifungal activity (even though they were less potent than fluconazole) [121].

**Figure 42:**
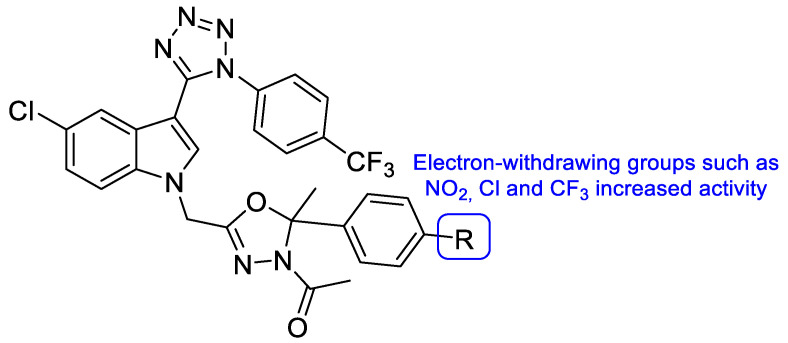
Chemical structure of the tetrazole-pyrazole compounds and SAR [107].

The pyrazole ring is an important structure in medicinal chemistry, since it is associated with a broad spectrum of therapeutical applications, such as analgetic, anti-inflammatory, antibacterial, antifungal, among others [122]. Therefore, various teams have tried to take advantage of this moiety to the search of new antifungal drugs. Tetrazole-pyrazole hybrids were synthesized and tested against *C. albicans* and *A. niger* for their antifungal activity. Although the results were not satisfactory, with DIZ values smaller than the standard ketoconazole, this study led to an interesting observation. The presence of electron-withdrawing groups in the phenyl group linked to the pyrazole moiety, such as chlorine, nitro, and trifluoromethyl, was beneficial to the antifungal activity while electron donating-groups, such as methoxyl and methyl, were detrimental to the activity (when compared to the unsubstituted analogs) (Figure 42) [107].

**Figure 43:**
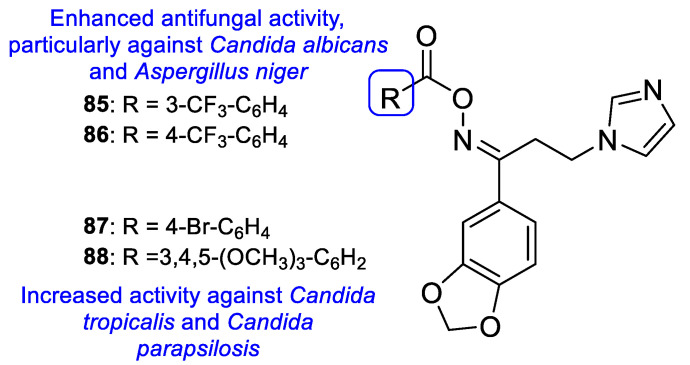
Chemical structures of compound **85**–**88**, and SAR [123].

Al-Wabli et al. synthesized benzodioxole-imidazole compounds (Figure 43) to assess their antifungal potential using the broth microdilution method to calculate MIC. These compounds were tested against four fungal strains: *C. albicans*, *C. tropicalis*, *C. parapsilosis*, and *A. niger*. Fluconazole was used as standard for *Candida* isolates and ketoconazole as standard for *A. niger*. The compounds tested were designed to contain an imidazole ring, bearing a propyl spacer. Since most available azoles have an ethyl spacer separating the azole ring and an aromatic ring instead of a propyl one, the authors decided to study the pharmaceutical potential of new imidazole derivatives. Along with that structural modification, the compounds’ structure contained 1,3-benzodioxole aromatic moieties, which might also enhance their antifungal activity. From the results, it can be inferred that compounds **85** and **86** (Figure 43), which contain a trifluoromethylphenyl moiety in C-3 and C-4 respectively, presented good values of MIC for all tested strains (with MIC values ranging from 148 to 297 µM), when compared to the values presented by the controls (MIC values ranging from 45 to 51 µM for fluconazole and 20 µM for ketoconazole). From those values, some conclusions about SAR can be extracted when comparing to the other derivatives: the trifluoromethyl moiety, in C-3 or C-4, seems to particularly enhance the antifungal potential of the benzodioxole-imidazole hybrids, in particular in *C. albicans* and *A. niger* strains [123]. For the fungal strains *C. tropicalis* and *C. parapsilosis*, compounds **87** and **88** presented the best pairing of results of DIZ and MIC (with MIC values of 289 and 145 µM, respectively, for compound **87**, and 565 and 141 µM, respectively, for compound **88** and DIZ values of 20 and 18 mm, respectively, for compound **87** and 21 and 19 mm, respectively, for compound **88**). These results suggest that the 4-bromophenyl and 3,4,5-trimethoxyphenyl moieties, respectively, increased the antifungal activity of the derivatives for these particular strains [123].

**Figure 44:**
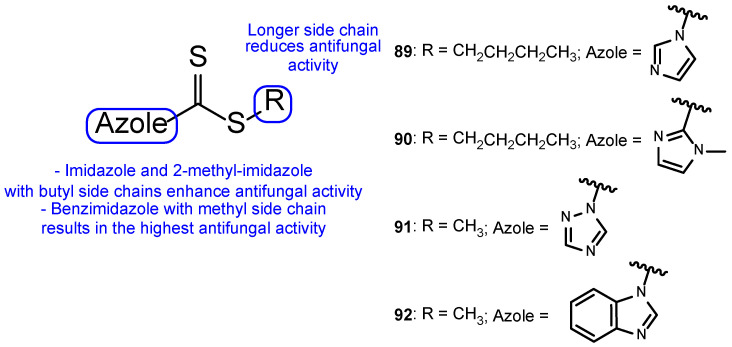
Chemical structure for compounds **89**–**92**, and SAR for the tested series of azole-carbodithioate hybrids [124].

In 2013, Kumar et al. studied a new series of azole-carbodithioate hybrids for their antifungal activity against various strains of *C. albicans*, using the broth microdilution method, and values were displayed as half maximal inhibitory concentration (IC_50_). Compounds **89**–**92** (Figure 44) revealed to be very promising, presenting interesting values of IC_50_, ranging from 6.05 to 239 µM (disregarding values >232 µM from compounds **90** and **91** for some of the strains). The most active compound of the series was **92**, with IC_50_ values from 6.05 to 16 µM (comparable to those of the standard drug fluconazole with IC_50_ = 0.42 and 0.69 µM against two of the tested strains, and no activity against the remaining strains). SAR studies revealed that increasing the side chain generally led to a reduced antifungal activity. Moreover, butyl side chains with imidazole and 2-methyl-imidazole displayed antifungal potential (compounds **89** and **90**, respectively). The combination of an imidazole ring fused to a benzene and a short length methyl side chain (compound **92**) was also revealed to enhance the antifungal activity against *C. albicans*. Nitro group in the azole ring was not a good substituent for the activity of this series of compounds (Figure 44). Additionally, compounds **89**–**92** were submitted to a cytotoxic assay against human cervical cell line (HeLa), where they demonstrated safety profiles, and to docking studies, where they were revealed to be promising leads for antifungal agents [124].

**Figure 45:**
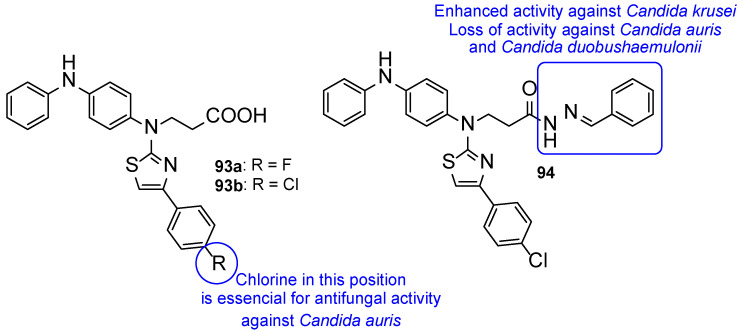
Chemical structure of compounds **93a**–**b** and **94**, as well as SAR for the *Candida* species [125].

A new series of compounds was synthesized by Malukaite et al., derived from thiazole and presenting β-amino acid and aromatic portions, and tested against azole-resistant *A. fumigatus* strains and against several multidrug-resistant *Candida* species. Compounds **93a** and **93b** (Figure 45) demonstrated good antifungal potential against *A. fumigatus*, with MIC values of 74 and 71 µM for the tested strains (excluding the wild type strain). Against *Candida* species, compounds **93b** and **94** (Figure 45) were the most promising ones, displaying antifungal activity against most of the tested strains. Compound **93b** was active against *C. auris* and *Candida duobushaemulonii*, with MIC values from 36 to 71 µM, but not against *C. krusei* or *C. albicans*. These data suggest that the chlorine substituent at C-4 on the phenyl ring is essential for the activity against *C. auris*. Further, **94**, with an additional phenyl substituent in the side chain, was active against *C. krusei* (MIC = 58 µM) but lost activity against *C. auris* and *C. duobushaemulonii*, suggesting that the addition of this ring led to a decrease in the antifungal activity (Figure 45). Cytotoxicity studies were performed using Vero cells, which revealed low cytotoxicity of the mentioned compounds, which makes them promising leads for the antifungal therapy [125].

### 4.4. Use of Triazoles in the Preparation of New Antifungals from Natural Products

Considering the importance of natural products in the design of new drug candidates and the increase in scope of click reactions, many studies have focused on the introduction of azole rings in these compounds, especially 1,2,3-triazole, in an attempt to improve their biological potential. A recent review highlighted this approach for a variety of pharmacological activities [126].

**Figure 46:**
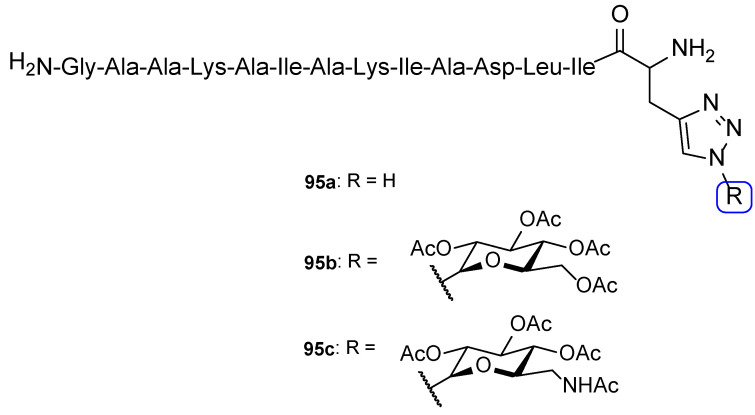
Chemical structures of **95a**–**c** [127].

Antimicrobial peptides have a broad spectrum of activities, which makes them interesting leads for pharmaceutical research and the development of new therapeutic drugs. Therefore, the scientific community has made modifications in the structures of these peptides to reach new derivatives with accentuated activity for antimicrobial-resistant microorganisms. Considering the antifungal activity, Junior et al. studied the synergic effect of triazole and saccharide rings, through different biophysical methods, by synthesizing and testing a series of glycotriazole-peptides for antifungal activity. Moreover, the effect of glycosylation in different fungal strains was assessed [127]. These triazole hybrids were synthesized from hylaseptin-P1 (HSP1), an antimicrobial peptide isolated from *Hyla punchata* anurans, with an amidated C-terminus (HSP1-NH_2_–GILDAIKAIAKAAG-NH_2_), observed to enhance the activity of several peptides. HSP1 was chosen to evaluate the effects of glycosylation and triazolation on the antifungal activity, as it is a compelling molecule for structural modifications. The glycosylation was conducted at the *N*-terminus, by substitution of the glycine-1 residue for a propargylglycine residue during the peptide chain synthesis [127]. The carboxyamidated C-terminus HSP1 (HSP1-NH_2_), the triazole-peptide derivative from HSP1-NH_2_ ([trz-G^1^]HSP1-NH_2_) (**95a**), the per-*O*-acetylated *N*-acetylglucose-triazole derivative from HSP1-NH_2_ ([p-Glc-trz-G^1^]HSP1-NH2) (**95b**), and the per-*O*-acetylated *N*-acetilglucosamine-triazole derivative from HSP1-NH_2_ ([p-GlcNAc-trz-G^1^]HSP1-NH_2_) (**95c**) (Figure 46) were tested against *C. tropicalis*, *C. parapsilosis*, and *C. krusei*. When tested against *Candida* spp. strains, the triazole derivatives presented notably higher antifungal activity (with MIC values ranging from 11.96 to 80.50 µM) than HSP1-NH_2_, which showed no activity (MIC values ranging from 136.90 to 149.90 µM). This suggests that the triazole moiety intensifies the peptide antifungal activity. Moreover, the glycotriazole-peptides **95b** and **95c** presented lower MIC values than **95a** and presented interesting values of MIC when compared to the antifungal drug 5-fluorocitosine, used as standard for the antifungal activity. These results suggest that both the triazole ring and carbohydrate ring lead to an enhanced antifungal activity of HSP1-NH_2_ [127].

**Figure 47:**
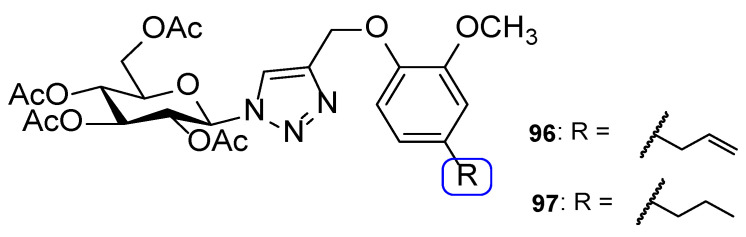
Chemical structures of compounds **96** and **97** [131].

Eugenol is a natural compound, presenting several therapeutical activities, with one of them being antifungal activity. Therefore, novel triazole molecules derived from eugenol were designed and synthesized by Magalhães et al. and tested against *C. albicans*, *C. tropicalis*, *C. krusei*, *C. glabrata*, and *C. parapsilosis* using the broth microdilution method. Compounds **96** and **97** (Figure 47) displayed the most promising antifungal activities (results were displayed in IC_50_). Compound **96**, with an allyl substituent, showed good values of IC_50_ against *C. tropicalis*, *C. krusei*, and *C. glabrata* (ranging from 26.1 to 52.1 µM) and compound **97**, with a propyl substituent, displayed good IC_50_ values against *C. tropicalis* and *C. krusei* (IC_50_ = 25.98 µM for both strains) and moderate activity against *C. glabrata* and *C. albicans* (IC_50_ = 103.94 and 173.2 µM, respectively), when compared to fluconazole values (IC_50_ ranging from 3.2 to 104.4 µM). SAR studies suggest that the presence of acetyl groups, combined with allyl (**96**) or propyl (**97**) groups, enhances the antifungal potency for these fungal strains. When compared to other compounds synthesized by the group in previous works (structures not shown), the analogs containing the triazole moiety revealed higher antifungal potency against *C. tropicalis* and *C. krusei* [128,129,130]. Moreover, the presence of the carbohydrate seems to be essential to the activity. Therefore, both these compounds are promising candidates for the treatment of *C. krusei*, since they were more effective and less toxic than the standard, fluconazole. In addition, the series of eugenol derived triazoles did not present relevant cytotoxic values when tested for cytotoxic activity in fibroblast cells from healthy human lung (MRC-5) using the MTT method. Docking studies (performed to all compounds) and molecular dynamics studies (performed to a strategic group of compounds), predicted compounds **96** and **97** to inhibit CYP51 by being transformed in vivo to their deacetylated analogs, acting as prodrugs [131].

The antifungal potential of compound **96** was also described one year later by Goswami et al. but against the opportunistic fungus *A. fumigatus* [132]. Compound **96** showed prominent antifungal activity with IC_50_ value of 5.42 μM. Moreover, the authors have found that this active compound possibly acts as inhibitor of cell wall-associated melanin hydrophobicity along with the number of conidia [132].

**Figure 48:**
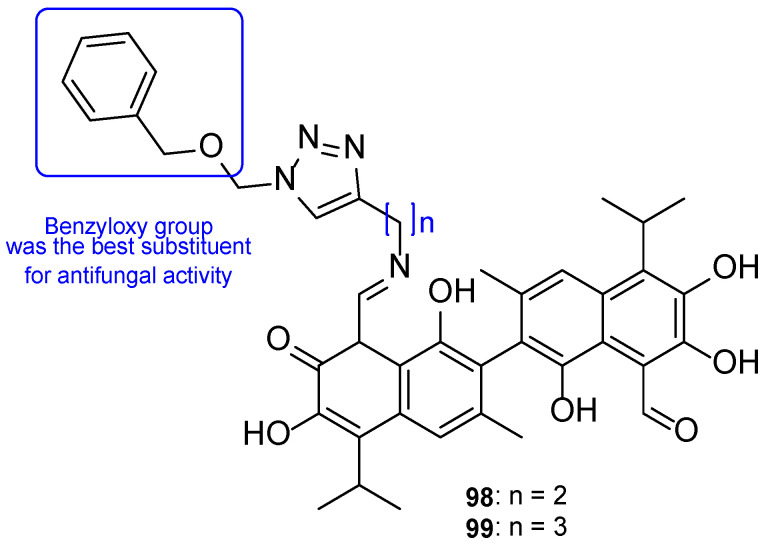
Chemical structures of compounds **98** and **99**, and SAR [133].

Pyta et al. synthesized 1,2,3-triazole conjugates from the natural product gossypol, a pigment present in cotton plants that is involved in defending them from insects and fungi. The 1,2,3-triazole was functionalized with aliphatic chains and benzyloxy groups and derivatives were tested against *F. oxysporum*. Two of these conjugates, compounds **98** and **99** (Figure 48), showed antifungal potency comparable to that of miconazole against *F. oxysporum* (MIC values of 0.022 µM). Moreover, the authors have demonstrated that the mechanism of action of these gossypol derivatives may be related to the inhibition of ergosterol biosynthesis [133].

**Figure 49:**
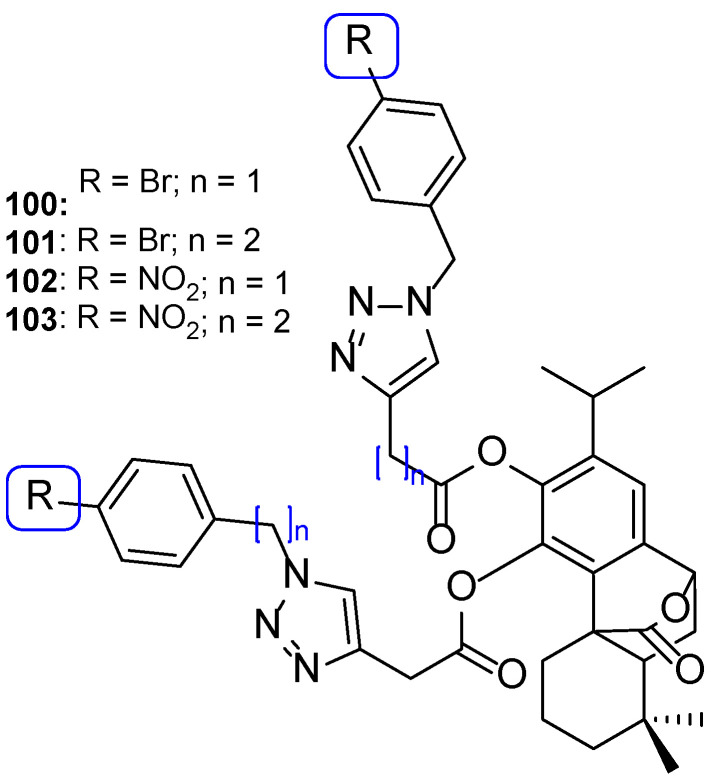
Chemical structure of compounds **100**–**103** [134].

In another work, Pertino et al. considered the synthesis of triazole derivatives from the natural product carnosol, which is a diterpene present in the leaves of *Rosmarinus officinalis* L. and that has many reported biological activities, including antifungal. They have prepared 24 new triazoles and the antifungal activity was checked as the percentage of inhibition of one strain of *C. albicans* and one strain of *C. neoformans* in the range of 250–3.9 µg/mL. The most active derivatives were **100**, **101**, **102**, and **103** (Figure 49), which were able to induce a decrease in fungal growth in the range of 91–71% at 250 µg/mL (282, 278, 305, and 300 µM, respectively for **100**, **101**, **102**, and **103**). Clearly, derivatives with a *p*-bromobenzyl or *p*-nitrobenzyl group were more active than the ones with an unsubstituted benzyl ring [134].

**Figure 50:**
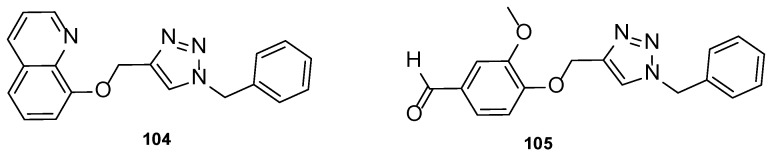
Chemical structure for compounds **104** and **105** [135].

In an attempt to develop antifungal agents with an innovative structural pattern, Irfan et al. synthesized hybrids between an *N*-benzyltriazole subunit and various hydroxylated natural products, such as vanillin, 8-hydroxyquinoline, eugenol, isoeugenol ferulic acid, and cuminol, among others. The most active compounds obtained were the triazoles linked to 8-hydroxyquinoline and vanillin (**104** and **105**, respectively, Figure 50). The results of in vitro antifungal activity showed that compound **104** was superior/comparable to fluconazole with IC_50_ values of 0.00014 µM against *C. albicans* and 0.011 µM against *C. tropicalis*. Compound **105** was the second most active with IC_50_ values of 0.00014 µM and 0.00029 µM, respectively, against the same strains. Of note, at their IC_50_ values, these compounds induced less than 5% hemolysis on human red blood cells, and compound **104** showed no cytotoxicity in VERO cells up to a concentration of 31.6 µM [135].

## 5. Conclusions

Azole compounds have been used for decades to treat fungal infections and are one of the most utilized classes of antifungal agents. However, researchers have been studying new strategies and techniques to obtain new antifungal drugs with improved safety and tolerability profiles, diminished drug–drug interactions, toxicity, and resistance problems, as well as enhanced antifungal potency. Various compounds have been designed, synthesized, and studied along the years, but given the structural diversity of the active compounds, it is difficult to generalize the nature of favorable substituents. Although the first generations of azoles had a well-known pharmacophore pattern, recent antifungal azole compounds show wider tolerance in structural variation. The emergence of tetrazole-containing compounds is also something to emphasize, since many new agents, e.g., VT-1161, VT-1129, and VT-1598, have been discovered with good profiles of safety, tolerability, and antimicrobial potency. Furthermore, a recent strategy is the introduction of azole moieties in bioactive natural products, which can lead to compounds with significative activity as antifungal agents, including against resistant strains.

Promising compounds in preclinical studies need further testing, such as toxicity studies, susceptibility evaluation against other species and mechanisms of action, as well as in vivo studies for a proof-of-concept. Moreover, studies with resistant strains are essential to determine the efficacy of these promising compounds in treating fungal conditions resistant (or becoming resistant) to the current arsenal of antifungal drugs. Moreover, SAR studies on new scaffolds would bring new perspectives and further improve lead optimization and the search for novel antifungals, while pharmacophore-based synthesis may help obtain efficient new leads.

## Figures and Tables

**Table 1:** Clinical trials for luliconazole (**1**) (summarized).

Phase	Identifier	Title	Condition(s)	Status	Ref.
4	NCT02394340	Study evaluating the drug interaction potential of luliconazole cream 1% in participants with tinea pedis and tinea cruris	Tinea pedis Tinea cruris	Completed	[33]
	NCT02767271	Maximal use of luliconazole cream 1% in pediatric participants with moderate to severe tinea pedis or tinea cruris	Tinea pedis Tinea cruris	Completed	[34]
4	NCT02767947	Safety and efficacy of product 33525 (luliconazole cream 1%) in pediatric participants with tinea corporis	Tinea corporis	Completed	[35]
2, 3	NCT01431820	Safety and efficacy of luliconazole solution, 10% in subjects with mild to moderate onychomycosis (solution)	Distal and lateral subungual onychomycosis	Completed	[36]
2	NCT00869336	Multicenter study of the efficacy and safety of luliconazole cream in tinea pedis (athlete’s foot)	Tinea pedis	Completed	[37]
1, 2	NCT01044381	Open-label pharmacokinetics/safety study of luliconazole solution, 10% in distal subungual onychomycosis	Onychomycosis	Completed	[38]
1	NCT05110638	Safety and tolerability study of SKX-16 (luliconazole 10% solution) in subjects with moderate to severe distal subungual onychomycosis	Onychomycosis of toenail	Active, not recruiting	[39]

**Table 2:** Mentioned clinical trials for isavuconazole (**3**) (summarized).

Phase	Identifier	Title	Condition(s)	Status	Ref.
3	NCT00412893	Isavuconazole (BAL8557) for primary treatment of invasive aspergillosis	Aspergillosis Invasive fungal infections	Completed	[48]
	NCT00634049	Isavuconazole in the treatment of renally impaired aspergillosis and rare fungi (vital)	Aspergillosis Invasive fungal infections	Completed	[49]
	NCT00413218	Isavuconazole (BAL8557) in the treatment of candidemia and other invasive *Candida* infections	Candidiasis, invasive Candidemia Mycoses	Completed	[50]

**Table 3:** Clinical trial for SUBA-itraconazole (summarized).

Phase	Identifier	Title	Condition(s)	Status	Ref.
3	NCT03572049	Endemic mycoses treatment with SUBA-itraconazole vs itraconazole (MSG15)	Invasive fungal infections	Completed	[54]

**Table 4:** Clinical trials for albaconazole (**8**) (summarized).

Phase	Identifier	Title	Condition(s)	Status	Ref.
2	NCT00730405	Efficacy and safety study of 4 dose regimens of oral albaconazole in subjects with distal subungual onychomycosis	Onychomycosis	Completed	[59]

**Table 5:** Clinical trials of PC945 (**16**) (summarized).

Phase	Identifier	Title	Condition(s)	Status	Ref.
3	NCT05238116	Safety and efficacy of PC945 in combination with other antifungal therapy for the treatment of refractory invasive pulmonary aspergillosis	Refractory IPA	Recruiting	[66]
2	NCT03905447	The effect of early treatment of PC945 on *Aspergillus fumigatus* lung infection in lung transplant patients	Aspergillosis Lung transplant infection	Terminated	[67]
	NCT03870841	The effect of PC945 on *Aspergillus fumigatus* lung infection in patients with cystic fibrosis	Aspergillosis Cystic fibrosis	Terminated	[68]
	NCT05037851	PC945 prophylaxis or pre-emptive therapy against pulmonary aspergillosis in lung transplant recipients	Pulmonary aspergillosis	Recruiting	[69]
	NCT03745196	The effect of PC945 on *Aspergillus* or *Candida* lung infections in patients with asthma or chronic respiratory diseases	Asthma Respiratory candidiasis Respiratory aspergillosis COPD Bronchiectasis	Terminated	[70]
1	NCT02715570	A study to investigate the safety, tolerability and pharmacokinetics of single and repeat doses of PC945	Aspergillosis	Completed	[71]

**Table 6:** Clinical trials for VT-1161 (**17**) and VT-1598 (**19**) (summarized).

Drug	Phase	Identifier	Title	Condition(s)	Status	Ref.
Oteseconazole (**17**)	3	NCT03562156; NCT03561701	A study of oral oteseconazole for the treatment of patients with recurrent vaginal candidiasis (yeast infection) (violet)	Recurrent Vaginal Candidiasis	Completed	[88,89]
		NCT03840616	Study of oral oteseconazole (VT-1161) for acute yeast infections in patients with recurrent yeast infections (ultraviolet)	Recurrent vaginal candidiasis	Completed	[90]
	2	NCT02267382	A study to evaluate oral VT-1161 in the treatment of patients with recurrent vaginal candidiasis (yeast infection)	Recurrent vaginal candidiasis	Completed	[77,91]
		NCT02267356	A study to evaluate the efficacy and safety of oral VT-1161 in patients with onychomycosis of the toenail	Onychomycosis	Completed	[92]
		NCT01891331	A study to evaluate the efficacy and safety of oral VT-1161 in patients with acute vaginal candidiasis (yeast infection)	Candidiasis, vulvovaginal	Completed	[93]
		NCT01891305	A study to evaluate the efficacy and safety of oral VT-1161 in patients with moderate—severe interdigital tinea pedis	Tinea pedis	Completed	[94]
VT-1598 (**19**)	1	NCT04208321	Safety and pharmacokinetics of VT-1598	Coccidioidomycosis	Completed	[95]

**Table 7:** Compilation of favorable substitutions and SAR for triazole and tetrazole molecules with a traditional azole pharmacophore.

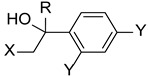			
X	Y	R	SAR
Triazole 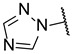	F	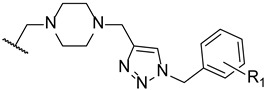	R_1_ = Halogens, methyl, cyanide, and nitro groups led to promising antifungal potential against *Candida albicans*, *Candida parapsilosis*, *Cryptococcus neoformans*, and *Nannizzia gypsea*
	F or Cl	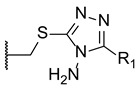	R_1_ = Di-chloro-phenyl, paired with Y = Cl, led to the highest antifungal potential of the tested series Y = Cl increased antifungal activity against *C. albicans*, *C. parapsilosis*, *C. neoformans*, *Epidermophyton floccosum* and *Trichophyton mentagrophytes*
	F	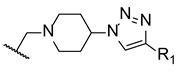	R_1_ = butyrate and butyric acid increased antifungal potential against all tested strains; 4-acetyl or 4-trifluoromethoxy-phenyl groups increased activity for *C. albicans*; 2-methyl-phenyl group increased activity against *C. parapsilosis* and *Candida glabrata*
	F or Cl	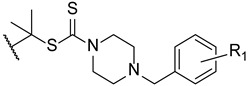	R_1_ = 4-Chloro showed high antifungal activity against *C. albicans*, *C. glabrata*, *C. parapsilosis*, *Candida krusei* and *Candida tropicalis*. Y = F analogs presented higher antifungal potency
	F	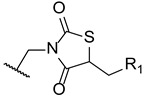	R_1_ = Di-chloro-phenyl, furan ring presented promising results against *C. albicans*
	F	1 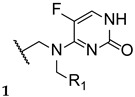	1: R_1_ = alkyl chains were not beneficial for antifungal activity 2: R_1–3_ = one fluor substituent; two chlorine substituents: increased antifungal activity against *C. albicans*
		2 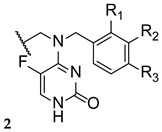	
	F	1 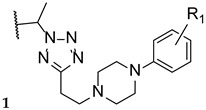	1 and 2: positional isomers R_1_ = trifluoromethyl led to high antifungal activity against *C. albicans*, *C. tropicalis*, *C. parapsilosis*, *C. krusei*, *C. glabrata*, *C. neoformans*, *Aspergillus fumigatus*, and *Aspergillus niger*
		2 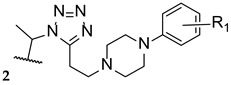	
Tetrazole 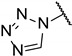	F	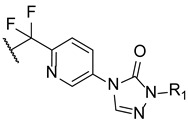	R_1_ = Alicyclic side chains up to six-members enhance the antifungal potency against *C. albicans*, *C. parapsilosis*, *C. glabrata*, *C. neoformans*, and *A. fumigatus*

## Data Availability

Not applicable.

## References

[1] Kainz K., Bauer M.A., Madeo F., Carmona-Gutierrez D. (2020). Fungal infections in humans: The silent crisis. Microb. Cell.

[2] Bongomin F., Gago S., Oladele R.O., Denning D.W. (2017). Global and Multi-National Prevalence of Fungal Diseases-Estimate Precision. J. Fungi.

[3] Kathiravan M.K., Salake A.B., Chothe A.S., Dudhe P.B., Watode R.P., Mukta M.S., Gadhwe S. (2012). The biology and chemistry of antifungal agents: A review. Bioorg. Med. Chem..

[4] Zhang H., Zhu A. (2020). Emerging Invasive Fungal Infections: Clinical Features and Controversies in Diagnosis and Treatment Processes. Infect. Drug Resist..

[5] Bouz G., Doležal M. (2021). Advances in Antifungal Drug Development: An Up-To-Date Mini Review. Pharmaceuticals.

[6] Campoy S., Adrio J.L. (2017). Antifungals. Biochem. Pharmacol..

[7] Stewart A.G., Paterson D.L. (2021). How urgent is the need for new antifungals?. Expert Opin. Pharmacother..

[8] Peyton L.R., Gallagher S., Hashemzadeh M. (2015). Triazole antifungals: A review. Drugs Today.

[9] Shafiei M., Peyton L., Hashemzadeh M., Foroumadi A. (2020). History of the development of antifungal azoles: A review on structures, SAR, and mechanism of action. Bioorg. Chem..

[10] Kuznetsov A. (2021). Introductory Chapter: Azoles, Their Importance, and Applications.

[11] Eicher T., Hauptmann S., Speicher A. (2013). The Chemistry of Heterocycles: Structures, Reactions, Synthesis, and Applications.

[12] Wadhwa P., Singh K. (2018). Multicomponent Reactions: A Sustainable Tool to 1,2- and 1,3-Azoles. Org. Biomol. Chem..

[13] Kerru N., Gummidi L., Maddila S., Jonnalagadda S. (2020). A Review of Recent Advances in the Green Synthesis of Azole- and Pyran-based Fused Heterocycles Using MCRs and Sustainable Catalysts. Curr. Org. Chem..

[14] Budeev A., Kantin G., Dar’in D., Krasavin M. (2021). Diazocarbonyl and Related Compounds in the Synthesis of Azoles. Molecules.

[15] Neha, Dwivedi A., Kumar R., Kumar V. (2017). Recent Synthetic Strategies for Monocyclic Azole Nucleus and Its Role in Drug Discovery and Development. Curr. Org. Synth..

[16] Siwach A., Verma P.K. (2021). Synthesis and therapeutic potential of imidazole containing compounds. BMC Chem..

[17] Woolley D.W. (1944). Some biological effects produced by benzimidazole and their reversal by purines. J. Biol. Chem..

[18] Sheehan D.J., Hitchcock C.A., Sibley C.M. (1999). Current and emerging azole antifungal agents. Clin. Microbiol. Rev..

[19] Maertens J.A. (2004). History of the development of azole derivatives. Clin. Microbiol. Infect..

[20] Chen S.C.A., Sorrell T.C. (2007). Antifungal agents. Med. J. Aust..

[21] Andes D., Dismukes W. (2011). Azoles. Essentials of Clinical Mycology.

[22] Kane A., Carter D.A. (2022). Augmenting Azoles with Drug Synergy to Expand the Antifungal Toolbox. Pharmaceuticals.

[23] Odds F.C., Brown A.J., Gow N.A. (2003). Antifungal agents: Mechanisms of action. Trends Microbiol..

[24] Delattin N., Cammue B.P., Thevissen K. (2014). Reactive oxygen species-inducing antifungal agents and their activity against fungal biofilms. Future Med. Chem..

[25] *S. cerevisiae* CYP51 Complexed with Fluconazole in the Active Site. https://www.rcsb.org/structure/4WMZ.

[26] Crystal Structure of Sterol 14-Alpha Demethylase (CYP51) from *Candida albicans* in Complex with the Tetrazole-Based Antifungal Drug Candidate VT1161 (VT1). https://www.rcsb.org/structure/5TZ1.

[27] Fisher M.C., Alastruey-Izquierdo A., Berman J., Bicanic T., Bignell E.M., Bowyer P., Bromley M., Brüggemann R., Garber G., Cornely O.A. (2022). Tackling the emerging threat of antifungal resistance to human health. Nat. Rev. Microbiol..

[28] Lepesheva G.I., Waterman M.R. (2007). Sterol 14alpha-demethylase cytochrome P450 (CYP51), a P450 in all biological kingdoms. Biochim. Biophys. Acta.

[29] Perfect J.R., Ghannoum M. (2020). Emerging Issues in Antifungal Resistance. Infect. Dis. Clin. N. Am..

[30] Khanna D., Bharti S. (2014). Luliconazole for the treatment of fungal infections: An evidence-based review. Core Evid..

[31] Niwano Y., Kuzuhara N., Kodama H., Yoshida M., Miyazaki T., Yamaguchi H. (1998). In Vitro and In Vivo Antidermatophyte Activities of NND-502, a Novel Optically Active Imidazole Antimycotic Agent. Antimicrob. Agents Chemother..

[32] Niwano Y., Kuzuhara N., Goto Y., Munechika Y., Kodama H., Kanai K., Yoshida M., Miyazaki T., Yamaguchi H. (1999). Efficacy of NND-502, a novel imidazole antimycotic agent, in experimental models of *Candida albicans* and *Aspergillus fumigatus* infections. Int. J. Antimicrob. Agents.

[33] Study Evaluating the Drug Interaction Potential of Luliconazole Cream 1% in Participants with Tinea Pedis and Tinea Cruris. https://www.clinicaltrials.gov/ct2/show/NCT02394340?term=luliconazole&draw=2&rank=1.

[34] Maximal Use of Luliconazole Cream 1% in Pediatric Participants with Moderate to Severe Tinea Pedis or Tinea Cruris. https://www.clinicaltrials.gov/ct2/show/NCT02767271?term=luliconazole&draw=2&rank=2.

[35] Safety and Efficacy of Product 33525 (Luliconazole Cream 1%) in Pediatric Participants with Tinea Corporis. https://www.clinicaltrials.gov/ct2/show/NCT02767947?term=luliconazole&draw=2&rank=3.

[36] Safety and Efficacy of Luliconazole Solution, 10% in Subjects with Mild to Moderate Onychomycosis (SOLUTION). https://www.clinicaltrials.gov/ct2/show/NCT01431820?term=luliconazole&draw=2&rank=6.

[37] Multicenter Study of the Efficacy and Safety of Luliconazole Cream in Tinea Pedis (Athlete’s Foot). https://www.clinicaltrials.gov/ct2/show/NCT00869336?term=luliconazole&draw=2&rank=7.

[38] Open-Label Pharmacokinetics/Safety Study of Luliconazole Solution, 10% in Distal Subungual Onychomycosis. https://www.clinicaltrials.gov/ct2/show/NCT01044381?term=luliconazole&draw=2&rank=5.

[39] Safety and Tolerability Study of SKX-16 (Luliconazole 10% Solution) in Subjects with Moderate to Severe Distal Subungual Onychomycosis. https://www.clinicaltrials.gov/ct2/show/NCT05110638?term=luliconazole&draw=2&rank=4.

[40] Ellsworth M., Ostrosky-Zeichner L. (2020). Isavuconazole: Mechanism of Action, Clinical Efficacy, and Resistance. J. Fungi.

[41] Guinea J., Peláez T., Recio S., Torres-Narbona M., Bouza E. (2008). In vitro antifungal activities of isavuconazole (BAL4815), voriconazole, and fluconazole against 1007 isolates of zygomycete, *Candida*, *Aspergillus*, *Fusarium*, and *Scedosporium* species. Antimicrob. Agents Chemother..

[42] Datta K., Rhee P., Byrnes E., Garcia-Effron G., Perlin D.S., Staab J.F., Marr K.A. (2013). Isavuconazole activity against *Aspergillus lentulus*, *Neosartorya udagawae*, and *Cryptococcus gattii*, emerging fungal pathogens with reduced azole susceptibility. J. Clin. Microbiol..

[43] Seifert H., Aurbach U., Stefanik D., Cornely O. (2007). In vitro activities of isavuconazole and other antifungal agents against *Candida* bloodstream isolates. Antimicrob. Agents Chemother..

[44] Bongomin F., Maguire N., Moore C.B., Felton T., Rautemaa-Richardson R. (2019). Isavuconazole and voriconazole for the treatment of chronic pulmonary aspergillosis: A retrospective comparison of rates of adverse events. Mycoses.

[45] Marty F.M., Ostrosky-Zeichner L., Cornely O.A., Mullane K.M., Perfect J.R., Thompson G.R., Alangaden G.J., Brown J.M., Fredricks D.N., Heinz W.J. (2016). Isavuconazole treatment for mucormycosis: A single-arm open-label trial and case-control analysis. Lancet Infect. Dis..

[46] Kullberg B.J., Viscoli C., Pappas P.G., Vazquez J., Ostrosky-Zeichner L., Rotstein C., Sobel J.D., Herbrecht R., Rahav G., Jaruratanasirikul S. (2019). Isavuconazole Versus Caspofungin in the Treatment of Candidemia and Other Invasive *Candida* Infections: The ACTIVE Trial. Clin. Infect. Dis..

[47] Pagano L., Cattaneo C., Quattrone M., Oberti M., Mazzitelli M., Trecarichi E.M. (2020). Isavuconazole-Animal Data and Clinical Data. J. Fungi.

[48] Isavuconazole (BAL8557) for Primary Treatment of Invasive Aspergillosis. https://clinicaltrials.gov/ct2/show/NCT00412893.

[49] Isavuconazole in the Treatment of Renally Impaired Aspergillosis and Rare Fungi (VITAL). https://www.clinicaltrials.gov/ct2/show/NCT00634049?term=NCT00634049&draw=2&rank=1.

[50] Isavuconazole (BAL8557) in the Treatment of Candidemia and Other Invasive Candida Infections. https://clinicaltrials.gov/ct2/show/NCT00413218.

[51] Abuhelwa A.Y., Foster D.J., Mudge S., Hayes D., Upton R.N. (2015). Population pharmacokinetic modeling of itraconazole and hydroxyitraconazole for oral SUBA-itraconazole and sporanox capsule formulations in healthy subjects in fed and fasted states. Antimicrob. Agents Chemother..

[52] Lindsay J., Sandaradura I., Wong K., Arthur C., Stevenson W., Kerridge I., Fay K., Coyle L., Greenwood M. (2017). Serum levels, safety and tolerability of new formulation SUBA-itraconazole prophylaxis in patients with haematological malignancy or undergoing allogeneic stem cell transplantation. J. Antimicrob. Chemother..

[53] Gintjee T.J., Donnelley M.A., Thompson G.R. (2020). Aspiring Antifungals: Review of Current Antifungal Pipeline Developments. J. Fungi.

[54] Endemic Mycoses Treatment with SUBA-Itraconazole vs Itraconazole (MSG15). https://www.clinicaltrials.gov/ct2/show/results/NCT03572049?term=NCT03572049&draw=2&rank=1.

[55] Wu L., Zhou K., Zong W., Chen Y., Sheng C. (2021). Single dose pharmacokinetics of topical iodiconazole creams in healthy Chinese volunteers. Xenobiotica.

[56] Sun N., Xie Y., Sheng C., Cao Y., Zhang W., Chen H., Fan G. (2013). In vivo pharmacokinetics and in vitro antifungal activity of iodiconazole, a new triazole, determined by microdialysis sampling. Int. J. Antimicrob. Agents.

[57] Jiang Z., Wang Y., Wang W., Wang S., Xu B., Fan G., Dong G., Liu Y., Yao J., Miao Z. (2013). Discovery of highly potent triazole antifungal derivatives by heterocycle-benzene bioisosteric replacement. Eur. J. Med. Chem..

[58] Sigurgeirsson B., van Rossem K., Malahias S., Raterink K. (2013). A phase II, randomized, double-blind, placebo-controlled, parallel group, dose-ranging study to investigate the efficacy and safety of 4 dose regimens of oral albaconazole in patients with distal subungual onychomycosis. J. Am. Acad. Dermatol..

[59] Efficacy and Safety Study of 4 Dose Regimens of Oral Albaconazole in Subjects with Distal Subungual Onychomycosis. https://www.clinicaltrials.gov/ct2/show/NCT00730405?term=NCT00730405&draw=2&rank=1.

[60] Ding Z., Ni T., Xie F., Hao Y., Yu S., Chai X., Jin Y., Wang T., Jiang Y., Zhang D. (2020). Design, synthesis, and structure-activity relationship studies of novel triazole agents with strong antifungal activity against *Aspergillus fumigatus*. Bioorg. Med. Chem. Lett..

[61] Colley T., Alanio A., Kelly S.L., Sehra G., Kizawa Y., Warrilow A.G.S., Parker J.E., Kelly D.E., Kimura G., Anderson-Dring L. (2017). In Vitro and In Vivo Antifungal Profile of a Novel and Long-Acting Inhaled Azole, PC945, on *Aspergillus fumigatus* Infection. Antimicrob. Agents Chemother..

[62] Jacobs S., Zagaliotis P., Walsh T. (2022). Novel antifungal agents in clinical trials version 2; peer review: 2 approved. F1000Research.

[63] Colley T., Sehra G., Daly L., Kimura G., Nakaoki T., Nishimoto Y., Kizawa Y., Strong P., Rapeport G., Ito K. (2019). Antifungal synergy of a topical triazole, PC945, with a systemic triazole against respiratory *Aspergillus fumigatus* infection. Sci. Rep..

[64] Kimura G., Nakaoki T., Colley T., Rapeport G., Strong P., Ito K., Kizawa Y. (2017). In Vivo Biomarker Analysis of the Effects of Intranasally Dosed PC945, a Novel Antifungal Triazole, on *Aspergillus fumigatus* Infection in Immunocompromised Mice. Antimicrob. Agents Chemother..

[65] Cass L., Murray A., Davis A., Woodward K., Albayaty M., Ito K., Strong P., Ayrton J., Brindley C., Prosser J. (2021). Safety and nonclinical and clinical pharmacokinetics of PC945, a novel inhaled triazole antifungal agent. Pharmacol. Res. Perspect..

[66] Safety and Efficacy of PC945 in Combination with Other Antifungal Therapy for the Treatment of Refractory Invasive Pulmonary Aspergillosis. https://www.clinicaltrials.gov/ct2/show/NCT05238116?term=PC-945&draw=2&rank=3.

[67] The Effect of Early Treatment of PC945 on *Aspergillus fumigatus* Lung Infection in Lung Transplant Patients. https://www.clinicaltrials.gov/ct2/show/NCT03905447?term=PC-945&draw=2&rank=2.

[68] The effect of PC945 on *Aspergillus fumigatus* Lung Infection in Patients with Cystic Fibrosis. https://www.clinicaltrials.gov/ct2/show/NCT03870841?term=PC-945&draw=2&rank=1.

[69] PC945 Prophylaxis or Pre-emptive Therapy against Pulmonary Aspergillosis in Lung Transplant Recipients. https://www.clinicaltrials.gov/ct2/show/NCT05037851?term=PC-945&draw=2&rank=4.

[70] The Effect of PC945 on Aspergillus or Candida Lung Infections in Patients with Asthma or Chronic Respiratory Diseases. https://www.clinicaltrials.gov/ct2/show/NCT03745196?term=PC-945&draw=2&rank=5.

[71] A Study to Investigate the Safety, Tolerability and Pharmacokinetics of Single and Repeat Doses of PC945. https://www.clinicaltrials.gov/ct2/show/NCT02715570?term=PC-945&draw=2&rank=6.

[72] Hoekstra W.J., Garvey E.P., Moore W.R., Rafferty S.W., Yates C.M., Schotzinger R.J. (2014). Design and optimization of highly-selective fungal CYP51 inhibitors. Bioorg. Med. Chem. Lett..

[73] Warrilow A.G., Hull C.M., Parker J.E., Garvey E.P., Hoekstra W.J., Moore W.R., Schotzinger R.J., Kelly D.E., Kelly S.L. (2014). The clinical candidate VT-1161 is a highly potent inhibitor of *Candida albicans* CYP51 but fails to bind the human enzyme. Antimicrob. Agents Chemother..

[74] Garvey E.P., Hoekstra W.J., Schotzinger R.J., Sobel J.D., Lilly E.A., Fidel P.L. (2015). Efficacy of the clinical agent VT-1161 against fluconazole-sensitive and -resistant *Candida albicans* in a murine model of vaginal candidiasis. Antimicrob. Agents Chemother..

[75] Shubitz L.F., Trinh H.T., Galgiani J.N., Lewis M.L., Fothergill A.W., Wiederhold N.P., Barker B.M., Lewis E.R., Doyle A.L., Hoekstra W.J. (2015). Evaluation of VT-1161 for Treatment of Coccidioidomycosis in Murine Infection Models. Antimicrob. Agents Chemother..

[76] Sobel J.D., Nyirjesy P. (2021). Oteseconazole: An advance in treatment of recurrent vulvovaginal candidiasis. Future Microbiol..

[77] Brand S.R., Degenhardt T.P., Person K., Sobel J.D., Nyirjesy P., Schotzinger R.J., Tavakkol A. (2018). A phase 2, randomized, double-blind, placebo-controlled, dose-ranging study to evaluate the efficacy and safety of orally administered VT-1161 in the treatment of recurrent vulvovaginal candidiasis. Am. J. Obstet. Gynecol..

[78] FDA Approves Mycovia Pharmaceuticals’ VIVJOA™ (oteseconazole), the First and Only FDA-Approved Medication for Recurrent Vulvovaginal Candidiasis (Chronic Yeast Infection). https://www.businesswire.com/news/home/20220428005301/en/FDA-Approves-Mycovia-Pharmaceuticals%E2%80%99-VIVJOA%E2%84%A2-oteseconazole-the-First-and-Only-FDA-Approved-Medication-for-Recurrent-Vulvovaginal-Candidiasis-Chronic-Yeast-Infection.

[79] Drugs@FDA: FDA-Approved Drugs. https://www.accessdata.fda.gov/scripts/cder/daf/index.cfm?event=overview.process&ApplNo=215888.

[80] Elewski B., Brand S., Degenhardt T., Curelop S., Pollak R., Schotzinger R., Tavakkol A. (2021). A phase II, randomized, double-blind, placebo-controlled, dose-ranging study to evaluate the efficacy and safety of VT-1161 oral tablets in the treatment of patients with distal and lateral subungual onychomycosis of the toenail. Br. J. Dermatol..

[81] Brand S.R., Sobel J.D., Nyirjesy P., Ghannoum M.A., Schotzinger R.J., Degenhardt T.P. (2021). A Randomized Phase 2 Study of VT-1161 for the Treatment of Acute Vulvovaginal Candidiasis. Clin. Infect. Dis..

[82] Warrilow A.G., Parker J.E., Price C.L., Nes W.D., Garvey E.P., Hoekstra W.J., Schotzinger R.J., Kelly D.E., Kelly S.L. (2016). The Investigational Drug VT-1129 Is a Highly Potent Inhibitor of *Cryptococcus* Species CYP51 but Only Weakly Inhibits the Human Enzyme. Antimicrob. Agents Chemother..

[83] Lockhart S.R., Fothergill A.W., Iqbal N., Bolden C.B., Grossman N.T., Garvey E.P., Brand S.R., Hoekstra W.J., Schotzinger R.J., Ottinger E. (2016). The Investigational Fungal Cyp51 Inhibitor VT-1129 Demonstrates Potent In Vitro Activity against *Cryptococcus neoformans* and *Cryptococcus gattii*. Antimicrob. Agents Chemother..

[84] Wiederhold N.P., Najvar L.K., Garvey E.P., Brand S.R., Xu X., Ottinger E.A., Alimardanov A., Cradock J., Behnke M., Hoekstra W.J. (2018). The Fungal Cyp51 Inhibitor VT-1129 Is Efficacious in an Experimental Model of Cryptococcal Meningitis. Antimicrob. Agents Chemother..

[85] Wiederhold N.P., Xu X., Wang A., Najvar L.K., Garvey E.P., Ottinger E.A., Alimardanov A., Cradock J., Behnke M., Hoekstra W.J. (2018). In Vivo Efficacy of VT-1129 against Experimental Cryptococcal Meningitis with the Use of a Loading Dose-Maintenance Dose Administration Strategy. Antimicrob. Agents Chemother..

[86] Gonzalez-Lara M.F., Sifuentes-Osornio J., Ostrosky-Zeichner L. (2017). Drugs in Clinical Development for Fungal Infections. Drugs.

[87] Garvey E.P., Sharp A.D., Warn P.A., Yates C.M., Atari M., Thomas S., Schotzinger R.J. (2020). The novel fungal CYP51 inhibitor VT-1598 displays classic dose-dependent antifungal activity in murine models of invasive aspergillosis. Med. Mycol..

[88] A Study of Oral Oteseconazole (VT-1161) for the Treatment of Patients with Recurrent Vaginal Candidiasis (Yeast Infection) (VIOLET). https://www.clinicaltrials.gov/ct2/show/NCT03561701?term=VT-1161&draw=2&rank=1.

[89] A Study of Oral Oteseconazole for the Treatment of Patients with Recurrent Vaginal Candidiasis (Yeast Infection) (VIOLET). https://www.clinicaltrials.gov/ct2/show/NCT03562156?term=VT-1161&draw=2&rank=7.

[90] Study of Oral Oteseconazole (VT-1161) for Acute Yeast Infections in Patients with Recurrent Yeast Infections (ultraVIOLET). https://www.clinicaltrials.gov/ct2/show/NCT03840616?term=VT-1161&draw=2&rank=3.

[91] A Study to Evaluate Oral VT-1161 in the Treatment of Patients with Recurrent Vaginal Candidiasis (Yeast Infection). https://www.clinicaltrials.gov/ct2/show/NCT02267382?term=VT-1161&draw=2&rank=2.

[92] A Study to Evaluate the Efficacy and Safety of Oral VT-1161 in Patients with Onychomycosis of the Toenail. https://www.clinicaltrials.gov/ct2/show/NCT02267356?term=VT-1161&draw=2&rank=4.

[93] A Study to Evaluate the Efficacy and Safety of Oral VT-1161 in Patients with Acute Vaginal Candidiasis (Yeast Infection). https://www.clinicaltrials.gov/ct2/show/NCT01891331?term=VT-1161&draw=2&rank=5.

[94] A Study to Evaluate the Efficacy and Safety of Oral VT-1161 in Patients with Moderate—Severe Interdigital Tinea Pedis. https://www.clinicaltrials.gov/ct2/show/NCT01891305?term=VT-1161&draw=2&rank=6.

[95] Safety and Pharmacokinetics of VT-1598. https://www.clinicaltrials.gov/ct2/show/NCT04208321?term=VT1598&draw=2&rank=1.

[96] Wang Y., Xu K., Bai G., Huang L., Wu Q., Pan W., Yu S. (2014). Synthesis and antifungal activity of novel triazole compounds containing piperazine moiety. Molecules.

[97] Hashemi S., Badali H., Faramarzi M., Samadi N., Afsarian M., Irannejad H., Emami S. (2014). Novel triazole alcohol antifungals derived from fluconazole: Design, synthesis, and biological activity. Mol. Divers..

[98] Jiang Z., Gu J., Wang C., Wang S., Liu N., Jiang Y., Dong G., Wang Y., Liu Y., Yao J. (2014). Design, synthesis and antifungal activity of novel triazole derivatives containing substituted 1,2,3-triazole-piperdine side chains. Eur. J. Med. Chem..

[99] Mahmoudi Y., Badali H., Hashemi S.M., Ansari M., Fakhim H., Fallah M., Shokrzadeh M., Emami S. (2019). New potent antifungal triazole alcohols containing N-benzylpiperazine carbodithioate moiety: Synthesis, in vitro evaluation and in silico study. Bioorg. Chem..

[100] Wu S., He X., Che X., Wang S., Liu Y., Jiang Y., Liu N., Dong G., Yao J., Miao Z. (2014). Inside Cover: From Antidiabetic to Antifungal: Discovery of Highly Potent Triazole-Thiazolidinedione Hybrids as Novel Antifungal Agents. ChemMedChem.

[101] Fang X.F., Li D., Tangadanchu V.K.R., Gopala L., Gao W.W., Zhou C.H. (2017). Novel potentially antifungal hybrids of 5-flucytosine and fluconazole: Design, synthesis and bioactive evaluation. Bioorg. Med. Chem. Lett..

[102] Upadhayaya R.S., Sinha N., Jain S., Kishore N., Chandra R., Arora S.K. (2004). Optically active antifungal azoles: Synthesis and antifungal activity of (2R,3S)-2-(2,4-difluorophenyl)-3-(5-[2-[4-aryl-piperazin-1-yl]-ethyl]-tetrazol-2-yl/1-yl)-1-[1,2,4]-triazol-1-yl-butan-2-ol. Bioorg. Med. Chem..

[103] Qian A., Zheng Y., Wang R., Wei J., Cui Y., Cao X., Yang Y. (2018). Design, synthesis, and structure-activity relationship studies of novel tetrazole antifungal agents with potent activity, broad antifungal spectrum and high selectivity. Bioorg. Med. Chem. Lett..

[104] Subhedar D.D., Shaikh M.H., Nawale L., Yeware A., Sarkar D., Khan F.A., Sangshetti J.N., Shingate B.B. (2016). Novel tetrazoloquinoline-rhodanine conjugates: Highly efficient synthesis and biological evaluation. Bioorg. Med. Chem. Lett..

[105] Kategaonkar A.H., Sadaphal S.A., Shelke K.F., Kategaonkar A.H., Shingate B.B., Shingare M.S. (2010). Synthesis and in vitro Antimicrobial Activity of New Ethyl 2-(Ethoxyphosphono)-1-cyano-2-(substituted tetrazolo [1,5-a]quinolin-4-yl)ethanoate Derivatives. Chin. J. Chem..

[106] Nikam M.D., Mahajan P.S., Damale M.G., Sangshetti J.N., Dabhade S.K., Shinde D.W., Gill C.H. (2015). Synthesis, molecular docking and biological evaluation of some novel tetrazolo [1,5-a]quinoline incorporated pyrazoline and isoxazoline derivatives. Med. Chem. Res..

[107] Wang S.Q., Wang Y.F., Xu Z. (2019). Tetrazole hybrids and their antifungal activities. Eur. J. Med. Chem..

[108] Dhayanithi V., Syed S.S., Kumaran K., Reguraman K., Sankar J., Ragavan R., Goud S.K., Kumari N.S., Pati H.N. (2011). Synthesis of selected 5-thio-substituted tetrazole derivatives and evaluation of their antibacterial and antifungal activities. J. Serb. Chem. Soc..

[109] Altıntop M.D., Kaplancıklı Z.A., Ciftçi G.A., Demirel R. (2014). Synthesis and biological evaluation of thiazoline derivatives as new antimicrobial and anticancer agents. Eur. J. Med. Chem..

[110] Ozkay Y., Tunalı Y., Karaca H., Işıkdağ I. (2011). Antimicrobial activity of a new combination system of benzimidazole and various azoles. Arch. Pharm..

[111] Mungra D.C., Patel M.P., Patel R.G. (2011). Microwave-assisted synthesis of some new tetrazolo [1,5-a]quinoline-based benzimidazoles catalyzed by p-TsOH and investigation of their antimicrobial activity. Med. Chem. Res..

[112] Vembu S., Pazhamalai S., Gopalakrishnan M. (2016). Synthesis, spectral characterization, and effective antifungal evaluation of 1H-tetrazole containing 1,3,5-triazine dendrimers. Med. Chem. Res..

[113] Shanmugapandiyan P., Atmakuru R. (2008). Synthesis and antimicrobial activity of 1-(benzothiazol-2′-yl)-5- phenyl-tetrazole. Asian J. Chem..

[114] Łukowska-Chojnacka E., Mierzejewska J., Milner-Krawczyk M., Bondaryk M., Staniszewska M. (2016). Synthesis of novel tetrazole derivatives and evaluation of their antifungal activity. Bioorg. Med. Chem..

[115] Kanakaraju S., Suresh L. (2015). Design, synthesis, in vitro antimicrobial and cytotoxic evaluation of novel 1,2,3-selena/thiadiazolyltetrazole derivatives. RSC Adv..

[116] Shaikh S.K.J., Kamble R.R., Somagond S.M., Devarajegowda H.C., Dixit S.R., Joshi S.D. (2017). Tetrazolylmethyl quinolines: Design, docking studies, synthesis, anticancer and antifungal analyses. Eur. J. Med. Chem..

[117] Antypenko L.M., Kovalenko S.I., Antypenko O.M., Katsev A.M., Achkasova O.M. (2013). Design and Evaluation of Novel Antimicrobial and Anticancer Agents among Tetrazolo [1,5-c]quinazoline-5-thione S-Derivatives. Sci. Pharm..

[118] Vembu S., Pavadai P., Gopalakrishnan M. (2014). Synthesis, in vitro antifungal and antitubercular evaluation of novel amino pyrimidines based tetrazole derivatives. J. Pharm. Res..

[119] Vembu S., Pavadai P., Gopalakrishnan M. (2014). Design, in silico molecular docking studies, synthesis, spectral characterization and in vitro antifungal evaluation of 1-(4-(1H-tetrazole-1-yl) phenyl)-3-arylprop-2-en-1-ones. Der Pharma Chem..

[120] Dofe V.S., Sarkate A.P., Kathwate S.H., Gill C.H. (2017). Synthesis, antimicrobial activity and anti-biofilm activity of novel tetrazole derivatives. Heterocycl. Commun..

[121] Nandha B., Hazra K., Chandra J.N., Nargund L.V.G., Pp R., Harish M.S., Puranik D. (2013). Synthesis of some new substituted fluoro benzimidazoles and their antimicrobial screening. Der Pharma Chem..

[122] Faria J.V., Vegi P.F., Miguita A.G.C., dos Santos M.S., Boechat N., Bernardino A.M.R. (2017). Recently reported biological activities of pyrazole compounds. Bioorg. Med. Chem..

[123] Al-Wabli R.I., Al-Ghamdi A.R., Ghabbour H.A., Al-Agamy M.H., Attia M.I. (2019). Synthesis, structure elucidation, and antifungal potential of certain new benzodioxole-imidazole molecular hybrids bearing ester functionalities. Drug Des. Devel. Ther..

[124] Kumar L., Lal N., Kumar V., Sarswat A., Jangir S., Bala V., Kumar L., Kushwaha B., Pandey A.K., Siddiqi M.I. (2013). Azole-carbodithioate hybrids as vaginal anti-Candida contraceptive agents: Design, synthesis and docking studies. Eur. J. Med. Chem..

[125] Malūkaitė D., Grybaitė B., Vaickelionienė R., Vaickelionis G., Sapijanskaitė-Banevič B., Kavaliauskas P., Mickevičius V. (2022). Synthesis of Novel Thiazole Derivatives Bearing β-Amino Acid and Aromatic Moieties as Promising Scaffolds for the Development of New Antibacterial and Antifungal Candidates Targeting Multidrug-Resistant Pathogens. Molecules.

[126] Guo H.-Y., Chen Z.-A., Shen Q.-K., Quan Z.-S. (2021). Application of triazoles in the structural modification of natural products. J. Enzym. Inhib. Med. Chem..

[127] Junior E.F.C., Guimarães C., Franco L.L., Alves R.J., Kato K.C., Martins H.R., de Souza Filho J.D., Bemquerer M.P., Munhoz V.H.O., Resende J.M. (2017). Glycotriazole-peptides derived from the peptide HSP1: Synergistic effect of triazole and saccharide rings on the antifungal activity. Amino Acids.

[128] de Souza T.B., Orlandi M., Coelho L.F.L., Malaquias L.C.C., Dias A.L.T., de Carvalho R.R., Silva N.C., Carvalho D.T. (2014). Synthesis and in vitro evaluation of antifungal and cytotoxic activities of eugenol glycosides. Med. Chem. Res..

[129] de Souza T.B., de Oliveira Brito K.M., Silva N.C., Rocha R.P., de Sousa G.F., Duarte L.P., Coelho L.F.L., Dias A.L.T., Veloso M.P., Carvalho D.T. (2016). New Eugenol Glucoside-based Derivative Shows Fungistatic and Fungicidal Activity against Opportunistic Candida glabrata. Chem. Biol. Drug Des..

[130] Hipólito T.M.M., Bastos G.T.L., Barbosa T.W.L., de Souza T.B., Coelho L.F.L., Dias A.L.T., Rodríguez I.C., dos Santos M.H., Dias D.F., Franco L.L. (2018). Synthesis, activity, and docking studies of eugenol-based glucosides as new agents against *Candida* sp.. Chem. Biol. Drug Des..

[131] Magalhães L.S., Reis A.C.C., Nakao I.A., Péret V.A.C., Reis R., Silva N.C., Dias A.L.T., Carvalho D.T., Dias D.F., Brandão G.C. (2021). Glucosyl-1,2,3-triazoles derived from eugenol and analogues: Synthesis, anti-Candida activity, and molecular modeling studies in CYP-51. Chem. Biol. Drug Des..

[132] Goswami L., Gupta L., Paul S., Vermani M., Vijayaraghavan P., Bhattacharya A.K. (2022). Design and synthesis of eugenol/isoeugenol glycoconjugates and other analogues as antifungal agents against *Aspergillus fumigatus*. RSC Med. Chem..

[133] Pyta K., Blecha M., Janas A., Klich K., Pecyna P., Gajecka M., Przybylski P. (2016). Synthesis, structure and antimicrobial evaluation of a new gossypol triazole conjugates functionalized with aliphatic chains and benzyloxy groups. Bioorg. Med. Chem. Lett..

[134] Pertino M.W., Theoduloz C., Butassi E., Zacchino S., Schmeda-Hirschmann G. (2015). Synthesis, Antiproliferative and Antifungal Activities of 1,2,3-Triazole-Substituted Carnosic Acid and Carnosol Derivatives. Molecules.

[135] Irfan M., Aneja B., Yadava U., Khan S.I., Manzoor N., Daniliuc C.G., Abid M. (2015). Synthesis, QSAR and anticandidal evaluation of 1,2,3-triazoles derived from naturally bioactive scaffolds. Eur. J. Med. Chem..

